# Supplementary material for: Multi-ancestry study of blood lipid levels identifies four loci interacting with physical activity
Source: Nat Commun. 2019 Jan 22;10:376. doi: 10.1038/s41467-018-08008-w (PMC6342931; doi:10.1038/s41467-018-08008-w)
Supplement: Supplementary file 1 — Supplementary Information [file 41467_2018_8008_MOESM1_ESM.pdf]

SUPPLEMENTARY INFORMATION

Multi-Ancestry Study of Blood Lipid Levels Identifies Four Loci Interacting with Physical Activity

Kilpeläinen et al.

Table of contents

**Supplementary Tables**	**p. 3-10**
**Supplementary Note 1**	**p. 11-18**
**Supplementary Note 2**	**p. 19-28**
**Supplementary Note 3**	**p. 29-34**
**Supplementary Note 4**	**p. 35-40**
**Supplementary References**	**p. 41-42**

Supplementary Tables

Supplementary Table 1: Sample Size of Stage 1 Studies

		HDL			LDL			TG		
**Ancestry**	**Study**	**N Inactive**	**N Active**	**N Total**	**N Inactive**	**N Active**	**N Total**	**N Inactive**	**N Active**	**N Total**
**African**	ARIC	619	2106	2725	558	1920	2478	571	1959	2530
	CARDIA	226	683	909	226	683	909	226	683	909
	CHS	320	403	723	312	390	702	314	393	707
	GENOA	226	527	753	194	461	655	197	463	660
	HABC	613	481	1094	610	474	1084	614	481	1095
	HANDLS	283	302	585	271	289	560	272	291	563
	HUFS	191	811	1002	190	810	1000	190	809	999
	HYPERGEN	290	939	1229	280	910	1190	283	913	1196
	JHS	834	1042	1876	826	1030	1856	834	1042	1876
	MESA	456	1135	1591	456	1131	1587	456	1135	1591
	WHI-SHARe	3337	4663	8000	3337	4663	8000	3337	4663	8000
	**Total**	**7395**	**13092**	**20487**	**7260**	**12761**	**20021**	**7294**	**12832**	**20126**
**Asian**	GenSalt	464	1349	1813	458	1332	1790	464	1349	1813
	MESA	239	509	748	239	499	738	239	509	748
	SCHS Cases	537	182	719	537	182	719	NA	NA	NA
	SCHS Controls	863	419	1282	864	419	1283	NA	NA	NA
	SP2-610	348	580	928	346	576	922	347	580	927
	SP2-1M	302	611	913	296	607	903	302	611	913
	**Total**	**2753**	**3650**	**6403**	**2740**	**3615**	**6355**	**1352**	**3049**	**4401**
**European**	AGES	984	1405	2389	984	1405	2389	984	1405	2389
	ARIC	2261	7185	9446	2173	6874	9047	2216	7026	9242
	CARDIA	408	1225	1633	406	1219	1625	407	1225	1632
	CHS	730	2231	2961	714	2184	2898	727	2212	2939
	GS-SFHS	320	5805	6125	NA	NA	NA	NA	NA	NA
	CROATIA-Vis	242	232	474	242	232	474	243	232	475
	CROATIA-Korcula	177	305	482	177	304	481	178	305	483
	ERF	660	1248	1908	649	1237	1886	660	1248	1908
	FAMHS	1835	1714	3549	1834	1713	3547	1835	1714	3549
	FHS	1589	5178	6767	1576	5136	6712	1589	5181	6770
	GENOA	162	949	1111	156	912	1068	160	934	1094
	GOLDN	193	623	816	193	623	816	193	623	816
	HABC	531	1103	1634	517	1083	1600	531	1104	1635
	HYPERGEN	268	972	1240	253	918	1171	265	962	1227
	MESA	565	2018	2583	565	1998	2563	565	2020	2585
	NEO	1376	4247	5623	1350	4196	5546	1376	4247	5623
	RS1	503	2452	2955	436	2256	2692	494	2417	2911
	RS2	382	1471	1853	376	1444	1820	380	1460	1840
	WGHS	6844	16068	22912	6844	16068	22912	4886	11657	16543
	WHI-WHIMS	1626	3437	5063	1626	3437	5063	1626	3437	5063

	WHI-GARNET	1307	2071	3378	1307	2071	3378	1307	2071	3378
	**Total**	**22963**	**61939**	**84902**	**22378**	**55310**	**77688**	**20622**	**51480**	**72102**
**Hispanic**	MESA	523	930	1453	523	909	1432	523	930	1453
	WHI-SHARe	1260	2036	3296	1260	2036	3296	1260	2036	3296
	**Total**	**1783**	**2966**	**4749**	**1783**	**2945**	**4728**	**1783**	**2966**	**4749**
**Brazilian**	BAEPENDI	383	520	903	380	516	896	383	520	903
	PELOTAS	663	2872	3535	NA	NA	NA	NA	NA	NA
	**Total**	**1046**	**3392**	**4438**	**380**	**516**	**896**	**383**	**520**	**903**
**All**	**Total**	**35940**	**85039**	**120979**	**34541**	**75147**	**109688**	**31434**	**70847**	**102281**

Supplementary Table 2: Genotyping and Imputation in Stage 1 Studies

Study	Ancestry	Genotyping Platform	Imputation Software
AGES	European	Illumina Hu370CNV	MaCH (ver. 1.0.16)
ARIC	European	Affymetrix 6.0	IMPUTE2
ARIC	African	Affymetrix 6.0	IMPUTE2
BAEPENDI	Brazilian	Genome-wide SNP Human Array 6.0 (Affymetrix 6.0)	SHAPEIT and IMPUTE2
PELOTAS	Brazilian	Illumina HumanOmni 2.5-8v1	IMPUTE2 (ver. 2.3.0)
CARDIA	European	Affymetrix 6.0	BEAGLE ver. 3.3.2
CARDIA	African	Affymetrix 6.0	MaCH/minimac
CHS	European	Illumina 370CNV (merged with ITMAT-Broad-CARe); Illumina iSELECT	MaCH/minimac
CHS	African	Illumina HumanOmni-Quad_v1 BeadChip	IMPUTE ver. 2.2.2
GS -SFHS	European	Illumina HumanOmniPlusExome	ShapeIt &Impute2
CROATIA-Vis	European	Illumina HumanHap 370 CNV Duochip	ShapeIt &Impute2
CROATIA-Korcula	European	Illlumina Infinium HumanHap 300 Bead chip	ShapeIt &Impute2
ERF	European	Illumina 6k, 318K, 350K and 610K; Affymetrix 250K	MaCH 1.0.18.c
FAMHS	European	Illumina HumMap 550K, Human 610 Quadv1, or Human 1M-Duov3	MaCH (ver. 1.0.16)
FHS	European	Affymetrix Nsp, Sty and 50K gene centric	MaCH/minimac
GENOA	European	Affymetrix 6.0 & Illumina 1M-Duo Bead Chip	IMPUTE2
GENOA	African	Affymetrix 6.0 & Illumina 1M-Duo Bead Chip	IMPUTE2
GenSalt	Asian	Affymetrix 6.0	MaCH/minimac
GOLDN	European	Affymetrix 6.0	MaCH (ver. 1.0.16)
HABC	European	Illumina HumanCoreExome BeadChip	MaCH (ver. 1.0.16)
HABC	African	Illumina HumanCoreExome BeadChip	MaCH (ver. 1.0.16)
HANDLS	African	Illumina 1M and 1Mduo arrays	MaCH/minimac
HUFS	African	Affymetrix 6.0	MACH-Admix
HYPERGEN	European	Affymetrix 5.0	MaCH/minimac
HYPERGEN	African	Affymetrix 6.0	MaCH/minimac
JHS	African	Affymetrix 6.0	MaCH (ver. 1.0.16)
MESA	African/Asian/European/Hispanic	Affymetrix 6.0	IMPUTE2
NEO	European	Illumina HumanCoreExome-24v1_A Beadchip	IMPUTE2
RS1	European	Illumina 550 (+duo), Illumina 610 quad	MaCH (ver. 1.0)
RS2	European	Illumina 550 duo	MaCH 1.0
SCHS Cases	Asian	Illumina Illumina Omni Zhonghua-8	IMPUTE2
SCHS Controls	Asian	Illumina Illumina Omni Zhonghua-8	IMPUTE2
SP2-610	Asian	Illumina610Quad	MaCH

SP2-1M	Asian	Illumina1Mduov3	MaCH (ver. 1.0.16)
WGHS	European	Illumina HumanHap 300 DuoPlus	MaCH (ver. 1.0.16)
WHI-WHIMS	European	HumanOmniExpressExome-8v1_B	MaCH (ver. 1.0.16)
WHI-GARNET	European	Illumina HumanOmni1-Quad v1-0 B	MaCH (ver. 1.0.16)
WHI-SHARe	African	Affymetrix 6.0	MaCH (ver. 1.0.16)
WHI-SHARe	Hispanic	Affymetrix 6.0	MaCH (ver. 1.0.16)

Supplementary Table 3: Sample Size of Stage 2 Studies

Ancestry	Study	HDL N Inactive	HDL N Active	HDL N Total	LDL N Inactive	LDL N Active	LDL N Total	TG N Inactive	TG N Active	TG N Total
**African**	GeneSTAR	174	671	845	171	665	836	174	669	843
	HRS	1305	339	1644	NA	NA	NA	NA	NA	NA
	CFS	160	125	285	160	124	284	160	125	285
	HYPERGEN-AXIOM	88	330	418	85	315	400	87	319	406
	JUPITER	1217	389	1606	1217	389	1606	1217	389	1606
	AADHS	159	427	586	155	417	572	159	427	586
	**Total**	**3103**	**2281**	**5384**	**1788**	**1910**	**3698**	**1797**	**1929**	**3726**
**Asian**	DF-TJ	186	1233	1419	186	1233	1419	186	1233	1419
	BES-610	226	260	486	227	260	487	224	258	482
	BES-Omniexpress	97	291	388	97	291	388	97	291	388
	RHS	724	1425	2149	724	1425	2149	724	1424	2148
	SMHS/SWHS	1985	163	2148	1985	163	2148	258	32	290
	**Total**	**3218**	**3372**	**6590**	**3219**	**3372**	**6591**	**1489**	**3238**	**4727**
**European**	AIRWAVE	573	13406	13979	NA	NA	NA	NA	NA	NA
	BRIGHT	323	850	1173	293	800	1093	323	845	1168
	CFS	85	168	253	78	165	243	86	168	254
	CoLaus	3486	1436	4922	3436	1417	4853	3486	1436	4922
	DESIR	324	370	694	324	370	694	324	370	694
	DHS	358	811	1169	328	769	1097	358	811	1169
	DRsEXTRA	313	915	1228	313	915	1228	313	915	1228
	EGCUT-OMNIEXPR.	201	871	1072	201	871	1072	161	719	880
	EGCUT-HUMAN370	65	611	676	65	611	676	6	82	88
	EPIC	7561	10667	18228	7561	10668	18229	7778	11078	18856
	FUSION CASE	271	780	1051	248	737	985	271	780	1051
	FUSION CONTROL	133	748	881	133	746	879	133	748	881
	GeneSTAR	171	1066	1237	168	1050	1218	172	1068	1240
	Glacier	1498	1720	3218	1255	1318	2573	2038	2271	4309
	GRAPHIC	38	558	596	38	558	596	38	558	596
	HRS	5941	960	6901	NA	NA	NA	NA	NA	NA
	INGI-CARL	NA	NA	NA	NA	NA	NA	115	306	421
	INGI-FVG	212	666	878	212	666	878	212	666	878
	JUPITER	4119	4278	8397	4119	4278	8397	4119	4278	8397
	KORA S3	1574	1473	3047	1571	1470	3041	136	110	246
	KORA S4	1901	1849	3750	1897	1848	3745	716	561	1277
	LBC1936	219	574	793	NA	NA	NA	NA	NA	NA
	Lifelines	4568	6533	11101	4541	6452	10993	4569	6533	11102
	METSIM	3171	5336	8507	3171	5335	8506	3171	5336	8507
	NESDA	364	2125	2489	361	2115	2476	364	2131	2495
	PREVEND	591	2313	2904	551	2205	2756	571	2242	2813

	SHEEPCASE	511	431	942	489	419	908	520	435	955
	SHEEPCONTROLS	565	728	1293	556	722	1278	570	731	1301
	TRAILS-Pop	43	923	966	43	923	966	43	923	966
	TWINGENE	1327	1981	3308	1308	1956	3264	1327	1981	3308
	YFS	536	1428	1964	536	1428	1964	536	1428	1964
	**Total**	**41042**	**66575**	**107617**	**33796**	**50812**	**84608**	**32456**	**49510**	**81966**
**Hispanic**	IRASFS	60	882	942	60	882	942	60	882	942
	SOL	2559	7920	10479	2518	7779	10297	2559	7921	10480
	**Total**	**2619**	**8802**	**11421**	**2578**	**8661**	**11239**	**2619**	**8803**	**11422**
**All**	**Total**	**49982**	**81030**	**131012**	**41381**	**64755**	**106136**	**38361**	**63480**	**101841**

Supplementary Table 4: Genotyping and Imputation in Stage 2 Studies

Study	Ancestry	Genotyping Platform	Imputation Software
AIRWAVE	European	Illumina HumanCoreExome- 12v1-1	Minimac3
BES-610	Asian	Illumina Human610-Quad Beadchips	MaCH
BES-Omniexpress	Asian	Illumina OmniExpress	MaCH
BRIGHT	European	Affymetrix GeneChip 500k array	MaCH/minimac
CFS	European	Illumina Omni	IMPUTE2
CFS	African	Affymetrix	MACH-ADMIX
CoLaus	European	Affymetrix Human Mapping 500K	minimac
DESIR	European	Illumina	ShapeIt / IMPUTE2
DF-TJ	Asian	Affymetrix 6.0	MaCH/minimac
DHS	European	Affymetrix 5.0	IMPUTE2
DRsEXTRA	European	Illumina Cardiometabochip	MaCH/minimac
EGCUT-OMNIEXPRESS	European	Illumina OmniExpress	IMPUTE2
EGCUT-HUMAN370CNV	European	Illumina HumanCNV370	IMPUTE2
EPIC	European	UKBioBank Axiom	ShapeIT, IMPUTE
FUSION CASE	European	Illumina HumanHap300	MaCH/minimac
FUSION CONTROL	European	Illumina HumanHap300	MaCH/minimac
GeneSTAR	European	Illumina 1M_v1C	IMPUTE2
GeneSTAR	African	Illumina 1M_v1C	IMPUTE2
Glacier	European	Illumina Cardiometabochip	NA
GRAPHIC	European	HumanOmniExpress-12v1	IMPUTE2
HRS	European	Illumina Omni2.5 Beadchip	IMPUTE2
HRS	African	Illumina Omni2.5 Beadchip	IMPUTE2
HYPERGEN	African	Affymetrix Axiom chips	MACH-ADMIX
INGI-CARL	European	Illumina 370K	IMPUTE2
INGI-FVG	European	Illumina 370K	IMPUTE2
IRASFS	Hispanic	Illumina OmniExpress+1S	IMPUTE2
JUPITER	European	Illumina Omni 1M Quad	MaCH/minimac
JUPITER	African	Illumina Omni 1M Quad	MaCH/minimac
KORA S3	European	Illumina Omni 2.5/Illumina Omni Express	IMPUTE v2.3.0
KORA S4	European	Affymetrix Axiom	IMPUTE v2.3.0

LBC1936	European	Illumian 610-Quadv1	MaCH/minimac
Lifelines	European	Illumina Cyto SNP12 v2	MaCH/minimac
METSIM	European	Illumina OmniExpress	MaCH/minimac
NESDA	European	Affymetrix 5.0, Affymetrix 6.0	MACH/minimac
AADHS	African	Illumina Omni5 array	IMPUTE2
PREVEND	European	Illumina Cyto SNP12 v2 array	Beagle 3.3.1
RHS	Asian	illumina 550K / Omni2.5M	Beagle 4 (r1399)
SHEEPCASE	European	Illumina Cardiometabochip	NA
SHEEPCONTROLS	European	Illumina Cardiometabochip	NA
SMHS/SWHS	Asian	Affymetrix 6.0; Illumina OmniExpress, 550, and 1M	MaCH/minimac
SOL	Hispanic	Illumina SOL HCHS Custom 15041502 B3 array	IMPUTE2
TRAILS-Pop	European	Illumina Cyto SNP12 v2	IMPUTE v2
TWINGENE	European	Illumina OmniExpress BeadChip	MaCH/minimac
YFS	European	Illumina 670k custom	IMPUTE2

Supplementary Note 1

STAGE 1 (GENOME-WIDE DISCOVERY) STUDY DESCRIPTIONS

AGES (Age Gene/Environment Susceptibility Reykjavik Study): The AGES Reykjavik study originally comprised a random sample of 30,795 men and women born in 1907-1935 and living in Reykjavik in 1967. A total of 19,381 people attended, resulting in a 71% recruitment rate. The study sample was divided into six groups by birth year and birth date within month. One group was designated for longitudinal follow up and was examined in all stages; another was designated as a control group and was not included in examinations until 1991. Other groups were invited to participate in specific stages of the study. Between 2002 and 2006, the AGESReykjavik study re-examined 5,764 survivors of the original cohort who had participated before in the Reykjavik Study.

ARIC (Atherosclerosis Risk in Communities): The ARIC study is a population-based prospective cohort study of cardiovascular disease sponsored by the National Heart, Lung, and Blood Institute (NHLBI). ARIC included 15,792 individuals, predominantly European American and African American, aged 45-64 years at baseline (1987-89), chosen by probability sampling from four US communities. Cohort members completed three additional triennial follow-up examinations, a fifth exam in 2011-2013, and a sixth exam in 2016-2017. The ARIC study has been described in detail previously[1].

Baependi Heart Study (Brazil): The Baependi Heart Study is an ongoing family-based cohort conducted in a rural town of the state of Minas Gerais. The study has enrolled approximate 2,200 individuals (over 10% of the town's adult population) and 10-year follow up period of longitudinal data. Briefly, probands were selected at random across 11 out of the 12 census districts in Baependi. After enrolment, the proband's first-degree (parents, siblings, and offspring), second-degree (half-siblings, grandparents/grandchildren, uncles/aunts, nephews/nieces, and double cousins), and third-degree (first cousins, great uncles/aunts, and great nephews/nieces) relatives, and his/her respective spouse's relatives resident both within Baependi (municipal and rural area) and surrounding towns were invited to participate. Only individuals age 18 and older were eligible to participate in the study. The study is conducted from a clinic/office in an easily accessible sector of the town, where the questionnaires were completed. A broad range of phenotypes ranging from cardiovascular, neurocognitive, psychiatric, imaging, physiologic and several layers of endophenotypes like metabolomics and lipidomics have been collected throughout the years Details about follow-up visits and available data can be found in the cohort profile paper[2]. DNA samples were genotyped using the Affymetrix 6.0 genechip. After quality control, the data were prephased using SHAPEIT and imputed using IMPUTE2 based on 1000 Genomes haplotypes.

CARDIA (Coronary Artery Risk Development in Young Adults): CARDIA is a prospective multicenter study with 5,115 adults Caucasian and African American participants of the age group 18-30 years, recruited from four centers at the baseline examination in 1985-1986. The recruitment was done from the total community in Birmingham, AL, from selected census tracts in Chicago, IL and Minneapolis, MN; and from the Kaiser Permanente health plan membership in Oakland, CA. The

details of the study design for the CARDIA study have been previously published[3]. Nine examinations have been completed since initiation of the study, respectively in the years 0, 2, 5, 7, 10, 15, 20, 25 and 30. Written informed consent was obtained from participants at each examination and all study protocols were approved by the institutional review boards of the participating institutions. All participants were asked to fast for 12 hours before each clinic visit. Serum and plasma blood samples were drawn from the antecubital vein and stored at −70°C until analyzed. Plasma total cholesterol, HDL-c, and triglyceride levels were measured using enzymatic methods; HDL-c levels were measured after dextran-sulfate-magnesium precipitation of other lipoproteins. LDL-c levels were estimated with the Friedewald equation for individuals with fasting triglyceride values less than 400 mg/dL. Baseline measures were used in this analyses.

CHS (Cardiovascular Health Study): CHS is a population-based cohort study of risk factors for cardiovascular disease in adults 65 years of age or older conducted across four field centers[4]. The original predominantly European ancestry cohort of 5,201 persons was recruited in 1989-1990 from random samples of the Medicare eligibility lists and an additional predominately African-American cohort of 687 persons was enrolled in 1992-93 for a total sample of 5,888. Blood samples were drawn from all participants at their baseline examination and DNA was subsequently extracted from available samples. European ancestry participants were excluded from the GWAS study sample due to prevalent coronary heart disease, congestive heart failure, peripheral vascular disease, valvular heart disease, stroke, or transient ischemic attack at baseline. After QC, genotyping was successful for 3271 European ancestry and 823 African-American participants. CHS was approved by institutional review committees at each site and individuals in the present analysis gave informed consent including consent to use of genetic information for the study of cardiovascular disease.

CROATIA-Korcula: The CROATIA-Korcula study is a family-based, cross-sectional study in the isolated island of Korcula that included 965 examinees aged 18-95. Blood samples were collected in 2007 along with many clinical and biochemical measures and lifestyle and health questionnaires.

CROATIA-Vis: The CROATIA-Vis study is a family-based, cross-sectional study in the isolated island of Vis that included 1,056 examinees aged 8-93. Blood samples were collected in 2003 and 2004 along with many clinical and biochemical measures and lifestyle and health questionnaires.

ERF (Erasmus Rucphen Family study): Erasmus Rucphen Family is a family based study that includes inhabitants of a genetically isolated community in the South-West of the Netherlands, studied as part of the Genetic Research in Isolated Population (GRIP) program. The goal of the study is to identify the risk factors in the development of complex disorders. Study population includes approximately 3,000 individuals who are living descendants of 22 couples who lived in the isolate between 1850 and 1900 and had at least six children baptized in the community church. All data were collected between 2002 and 2005. All participants gave informed consent, and the Medical Ethics Committee of the Erasmus University Medical Centre approved the study.

FamHS (Family Heart Study): The NHLBI FamHS study design, collection of phenotypes and covariates as well as clinical examination have been previously described[5]. In brief, the FamHS recruited 1,200 families (approximately 6,000 individuals), half randomly sampled, and half selected because of an excess of coronary heart disease (CHD) or risk factor abnormalities as compared with

age- and sex-specific population rates. The participants were sampled from four population-based parent studies: the Framingham Heart Study, the Utah Family Tree Study, and two centers for the Atherosclerosis Risk in Communities study (ARIC: Minneapolis, and Forsyth County, NC). These individuals attended a clinic exam (1994-1996) and a broad range of phenotypes were assessed in the general domains of CHD, atherosclerosis, cardiac and vascular function, inflammation and hemostasis, lipids and lipoproteins, blood pressure, diabetes and insulin resistance, pulmonary function, diet, education, socioeconomic status, habitual behavior, physical activity, anthropometry, medical history and medication use. Approximately 8 years later, study participants belonging to the largest pedigrees were invited for a second clinical exam (2002-04). The most important CHD risk factors were measured again, including lipids, parameters of glucose metabolism, blood pressure, anthropometry, and several biochemical and hematologic markers. In addition, a computed tomography examination provided measures of coronary and aortic calcification, and abdominal and liver fat burden. Medical history and medication use was updated. A total of 2,756 European ancestry subjects in 510 extended random and high CHD risk families were studied. Also, 633 African ancestry subjects were recruited at ARIC field center at the University of Alabama in Birmingham. Informed consent was obtained from all participants.

FHS (Framingham Heart Study): FHS began in 1948 with the recruitment of an original cohort of 5,209 men and women (mean age 44 years; 55 percent women). In 1971 a second generation of study participants was enrolled; this cohort (mean age 37 years; 52% women) consisted of 5,124 children and spouses of children of the original cohort. A third generation cohort of 4,095 children of offspring cohort participants (mean age 40 years; 53 percent women) was enrolled in 2002-2005 and are seen every 4 to 8 years. Details of study designs for the three cohorts are summarized elsewhere.

GENOA (Genetic Epidemiology Network of Arteriopathy): GENOA is one of four networks in the NHLBI Family-Blood Pressure Program (FBPP)[6,7]. GENOA's long-term objective is to elucidate the genetics of target organ complications of hypertension, including both atherosclerotic and arteriolosclerotic complications involving the heart, brain, kidneys, and peripheral arteries. The longitudinal GENOA Study recruited European-American and African-American sibships with at least 2 individuals with clinically diagnosed essential hypertension before age 60 years. All other members of the sibship were invited to participate regardless of their hypertension status. Participants were diagnosed with hypertension if they had either 1) a previous clinical diagnosis of hypertension by a physician with current anti-hypertensive treatment, or 2) an average systolic blood pressure ≥ 140 mm Hg or diastolic blood pressure ≥ 90 mm Hg based on the second and third readings at the time of their clinic visit. Exclusion criteria were secondary hypertension, alcoholism or drug abuse, pregnancy, insulin-dependent diabetes mellitus, or active malignancy. During the first exam (1995-2000), 1,583 European Americans from Rochester, MN and 1,854 African Americans from Jackson, MS were examined. Between 2000 and 2005, 1,241 of the European Americans and 1,482 of the African Americans returned for a second examination. Because African-American probands for GENOA were recruited through the Atherosclerosis Risk in Communities (ARIC) Jackson field center participants, we excluded ARIC participants from analyses.

GenSalt (Genetic Epidemiology Network of Salt Sensitivity): GenSalt is a multi-center, family based study designed to identify, through dietary sodium and potassium intervention, salt-sensitivitivity susceptibility genes which may underlie essential hypertension in rural Han Chinese families.

Approximately 629 families with at least one 'proband' with high blood pressure were recruited and tested for a wide variety of physiological, metabolic and biochemical measures at baseline and at multiple times during the 3-week intervention. The intervention consisted of one week on a low sodium diet, followed by one week on a high sodium diet, and finally one week on a high sodium diet with a potassium supplement.

GS:SFHS (Generation Scotland: Scottish Family Health Study): The GS:SFHS (www.generationscotland.org) is a family-based genetic epidemiology cohort with DNA, other biological samples (serum, urine and cryopreserved whole blood) and socio-demographic and clinical data from approximately 24,000 volunteers, aged 18-98 years, in ~7,000 family groups. An important feature of GS:SFHS is the breadth of phenotype information, including detailed data on cognitive function, personality traits and mental health. Although data collection was cross-sectional, GS:SFHS becomes a longitudinal cohort as a result of the ability to link to routine NHS data, using the community health index (CHI) number.

HANDLS (Healthy Aging in Neighborhoods of Diversity across the Life Span): HANDLS is a community-based, longitudinal epidemiologic study examining the influences of race and socioeconomic status (SES) on the development of age-related health disparities among a sample of socioeconomically diverse African Americans and whites. This unique study will assess over a 20-year period physical parameters and also evaluate genetic, biologic, demographic, and psychosocial, parameters of African American and white participants in higher and lower SES to understand the driving factors behind persistent black-white health disparities in overall longevity, cardiovascular disease, and cognitive decline. The study recruited 3,722 participants from Baltimore, MD with a mean age of 47.7 years, 2,200 African Americans and 1,522 whites, with 41% reporting household incomes below the 125% poverty delimiter.

Genotyping was done on a subset of self-reporting African American participants by the Laboratory of Neurogenetics, National Institute on Aging, National Institutes of Health (NIH). A larger genotyping effort included a small subset of self-reporting European ancestry samples. This research was supported by the Intramural Research Program of the NIH, NIA and the National Center on Minority Health and Health Disparities.

Health ABC (Health, Aging, and Body Composition): Cohort description: The Health ABC study is a prospective cohort study investigating the associations between body composition, weight-related health conditions, and incident functional limitation in older adults. Health ABC enrolled well-functioning, community-dwelling black (n=1281) and white (n=1794) men and women aged 70-79 years between April 1997 and June 1998. Participants were recruited from a random sample of white and all black Medicare eligible residents in the Pittsburgh, PA, and Memphis, TN, metropolitan areas. Participants have undergone annual exams and semi-annual phone interviews. The current study sample consists of 1559 white participants who attended the second exam in 1998-1999 with available genotyping data. Genotyping was performed by the Center for Inherited Disease Research (CIDR) using the Illumina Human1M-Duo BeadChip system.

HUFS (Howard University Family Study): HUFS followed a population-based selection strategy designed to be representative of African American families living in the Washington, DC metropolitan

area. The major objectives of the HUFS were to study the genetic and environmental basis of common complex diseases including hypertension, obesity and associated phenotypes. Participants were sought through door-to-door canvassing, advertisements in local print media and at health fairs and other community gatherings. In order to maximize the utility of this cohort for the study of multiple common traits, families were not ascertained based on any phenotype. During a clinical examination, demographic information was collected by interview.

HyperGEN (Hypertension Genetic Epidemiology Network): HyperGEN is a family-based study that looks at the genetic causes of hypertension and related conditions in EA and AA subjects. HyperGEN recruited hypertensive sibships, along with their normotensive adult offspring, and an age-matched random sample. HyperGEN has collected data on 2,471 Caucasian-American subjects and 2,300 African-American subjects, from five field centers in Alabama, Massachusetts, Minnesota, North Carolina, and Utah.

JHS (Jackson Heart Study): The Jackson Heart Study is a longitudinal, community-based observational cohort study investigating the role of environmental and genetic factors in the development of cardiovascular disease in African Americans[8-10]. Between 2000 and 2004, a total of 5306 participants were recruited from a tri-county area (Hinds, Madison, and Rankin Counties) that encompasses Jackson, MS. Details of the design and recruitment for the Jackson Heart Study cohort has been previously published.1-3 Briefly, approximately 30% of participants were former members of the Atherosclerosis Risk in Communities (ARIC) study. The remainder were recruited by either 1) random selection from the Accudata list, 2) commercial listing, 3) a constrained volunteer sample, in which recruitment was distributed among defined demographic cells in proportions designed to mirror those in the overall population, or through the Jackson Heart Study Family Study.

MESA (Multi-Ethnic Study of Atherosclerosis): The Multi-Ethnic Study of Atherosclerosis (MESA) is a study of the characteristics of subclinical cardiovascular disease and the risk factors that predict progression to clinically overt cardiovascular disease or progression of the subclinical disease[11]. MESA consisted of a diverse, population-based sample of an initial 6,814 asymptomatic men and women aged 45-84. 38 percent of the recruited participants were white, 28 percent African American, 22 percent Hispanic, and 12 percent Asian, predominantly of Chinese descent. Participants were recruited from six field centers across the United States: Wake Forest University, Columbia University, Johns Hopkins University, University of Minnesota, Northwestern University and University of California - Los Angeles. Participants are being followed for identification and characterization of cardiovascular disease events, including acute myocardial infarction and other forms of coronary heart disease (CHD), stroke, and congestive heart failure; for cardiovascular disease interventions; and for mortality. The first examination took place over two years, from July 2000 - July 2002. It was followed by four examination periods that were 17-20 months in length. Participants have been contacted every 9 to 12 months throughout the study to assess clinical morbidity and mortality.

NEO (The Netherlands Epidemiology of Obesity study): The NEO was designed for extensive phenotyping to investigate pathways that lead to obesity-related diseases. The NEO study is a population-based, prospective cohort study that includes 6,671 individuals aged 45–65 years, with an oversampling of individuals with overweight or obesity. At baseline, information on demography, lifestyle, and medical history have been collected by questionnaires. In addition, samples of 24-h urine,

fasting and postprandial blood plasma and serum, and DNA were collected. Genotyping was performed using the Illumina HumanCoreExome BeadChip, which was subsequently imputed to the 1000 genome reference panel. Participants underwent an extensive physical examination, including anthropometry, electrocardiography, spirometry, and measurement of the carotid artery intima-media thickness by ultrasonography. In random subsamples of participants, magnetic resonance imaging of abdominal fat, pulse wave velocity of the aorta, heart, and brain, magnetic resonance spectroscopy of the liver, indirect calorimetry, dual energy X-ray absorptiometry, or accelerometry measurements were performed. The collection of data started in September 2008 and completed at the end of September 2012. Participants are currently being followed for the incidence of obesity-related diseases and mortality.

Pelotas Birth Cohort Study (The 1982 Pelotas Birth Cohort Study, Brazil): The maternity hospitals in Pelotas, a southern Brazilian city (current population ~330,000), were visited daily in the year of 1982. The 5,914 liveborns whose families lived in the urban area were examined and their mothers interviewed. Information was obtained for more than 99% of the livebirths. These subjects have been followed-up at the following mean ages: 11.3 months (all children born from January to Abril 1982; n=1457), 19.4 months (entire cohort; n=4934), 43.1 months (entire cohort; n=4742), 13.1 years (random subsample; n=715), 14.7 years (systematic subsample; n=1076); 18.2 (male cohorts attending to compulsory Army recruitment examination; n=2250), 18.9 (systematic subsample; n=1031), 22.8 years (entire cohort; n=4297) and 30.2 years (entire cohort; n=3701). Details about follow-up visits and available data can be found in the two Cohort Profile papers[12-13]. DNA samples (collected at the mean age of 22.8 years) were genotyped for ~2.5 million of SNPs using the Illumina HumanOmni2.5-8v1 array (which includes autosomal, X and Y chromosomes, and mitochondrial variants). After quality control, the data were prephased using SHAPEIT and imputed using IMPUTE2 based on 1000 Genomes haplotypes.

RS (Rotterdam Study): The Rotterdam Study is a prospective, population-based cohort study among individuals living in the well-defined Ommoord district in the city of Rotterdam in The Netherlands. The aim of the study is to determine the occurrence of cardiovascular, neurological, ophthalmic, endocrine, hepatic, respiratory, and psychiatric diseases in elderly people. The cohort was initially defined in 1990 among approximately 7,900 persons, aged 55 years and older, who underwent a home interview and extensive physical examination at the baseline and during follow-up rounds every 3-4 years (RS-I). Cohort was extended in 2000/2001 (RS-II, 3,011 individuals aged 55 years and older) and 2006/2008 (RS-III, 3,932 subjects, aged 45 and older). Written informed consent was obtained from all participants and the Medical Ethics Committee of the Erasmus Medical Center, Rotterdam, approved the study.

SCHS-CHD (Singapore Chinese Health Study - Coronary Heart Disease): SCHS-CHD is a case-control study of coronary heart disease that was nested within the Singapore Chinese Health Study (SCHS), a prospective cohort study of 63,257 Singaporean Chinese men and women aged 45-74 years living in Singapore. We selected cases and controls from participants that provided blood samples and were free of coronary heart disease and stroke at the time of blood collection (N=24,454). Cases (N=760) had acute myocardial infarction (AMI) or died of coronary heart disease. AMI was identified through the Singapore Myocardial Infarction Registry or through the nationwide hospital discharge database followed by confirmation of AMI by cardiologists' review of medical records using the Multi-

Ethnic Study of Atherosclerosis criteria (available at: http://www.mesa-nhlbi.org/manuals.aspx). Coronary heart disease deaths were identified through the Singapore Registry of Births and Deaths (ICD9 410-414 as first stated cause of death). Matched controls (N=1,491) were selected using a risk-set sampling strategy. Controls were participants who were alive and free of coronary heart disease at the time of the diagnosis or death of the index cases and were matched for age, sex, dialect group, year of recruitment and date of blood collection. In-person interviews and phlebotomy were conducted before the onset of disease and non-fasting venous blood was stored at -80^{0}C for extraction of DNA and blood biochemistry.

Singapore: SP2 (Singapore Prospective Study Program): The SP2 is a population-based study of diabetes and cardiovascular disease in Singapore. It first surveyed subjects (Chinese, Malay and Indian) from four cross-sectional studies that were conducted in Singapore between 1982 and 1998. Subjects were between the ages of 24-95 years and represented a random sample of the Singapore population. Subjects were re-visited between 2003 and 2007. Among the 10,747 individuals who were eligible, 5,157 subjects completed a questionnaire and the subsequent clinical examinations. Of the 5,517 subjects, 2,434 Chinese were genotyped on a combination of Illumina 610, 1M and 550 arrays. Fasting HDL-C, TC and TG were measured by an automated analyzer autoanalyzer (ADVIA 2400, Bayer Diagnostics). LDL-C was calculated from Friedewald formula. Participants completed both the physical activity questionnaire in SP2 (SP2PAQ) and IPAQ long form[14]. Data from this re-visit were utilized for this study[15,16].

WGHS (Women's Genome Health Study): WGHS is a prospective cohort of female North American health care professionals representing participants in the Women's Health Study (WHS) trial who provided a blood sample at baseline and consent for blood-based analyses. Participants in the WHS were 45 years or older at enrollment and free of cardiovascular disease, cancer or other major chronic illness. The current data are derived from 23,294 WGHS participants for whom whole genome genotype information was available at the time of analysis and for whom self-reported European ancestry could be confirmed by multidimensional scaling analysis of 1,443 ancestry informative markers in PLINK v. 1.06. At baseline, lifestyle habits related to smoking, consumption of alcohol, and physical activity as well as other general clinical information were ascertained by a self-reported questionnaire, an approach which has been validated in the WGHS demographic, namely female health care professionals.

WHI (Women's Health Initiative): WHI is a long-term national health study that focuses on strategies for preventing common diseases such as heart disease, cancer and fracture in postmenopausal women. A total of 161,838 women aged 50–79 years old were recruited from 40 clinical centers in the US between 1993 and 1998. WHI consists of an observational study, two clinical trials of postmenopausal hormone therapy (HT, estrogen alone or estrogen plus progestin), a calcium and vitamin D supplement trial, and a dietary modification trial[17]. Study recruitment and exclusion criteria have been described previously[17]. Recruitment was done through mass mailing to age-eligible women obtained from voter registration, driver's license and Health Care Financing Administration or other insurance list, with emphasis on recruitment of minorities and older women[18]. Exclusions included participation in other randomized trials, predicted survival < 3 years, alcoholism, drug dependency, mental illness and dementia. For the CT, women were ineligible if they had a systolic BP > 200 mm Hg or diastolic BP > 105 mm Hg, a history of hypertriglyceridemia or breast cancer. Study protocols and

consent forms were approved by the IRB at all participating institutions. Socio-demographic characteristics, lifestyle, medical history and self-reported medications were collected using standardized questionnaires at the screening visit. Physical measures of height, weight and blood pressure were measured at a baseline clinical visit[18]. The genome wide association study (GWAS) non-overlapping samples are composed of a case-control study (WHI Genomics and Randomized Trials Network – GARNET, which included all coronary heart disease, stroke, venous thromboembolic events and selected diabetes cases that happened during the active intervention phase in the WHI HT clinical trials and aged matched controls), women selected to be "representative" of the HT trial (mostly younger white HT subjects that were also enrolled in the WHI memory study - WHIMS) and the WHI SNP Health Association Resource (WHI SHARe), a randomly selected sample of 8,515 African American and 3,642 Hispanic women from WHI. GWAS was performed using Affymetrix 6.0 (WHI-SHARe), HumanOmniExpressExome-8v1_B (WHIMS), Illumina HumanOmni1-Quad v1-0 B (GARNET). Extensive quality control (QC) of the GWAS data included alignment ("flipping") to the same reference panel, imputation to the 1000G data (using the recent reference panel - v3.20101123), identification of genetically related individuals, and computations of principal components (PCs) using methods developed by Price et al. (using EIGENSOFT software 53), and finally the comparison with self-reported ethnicity. After QC and exclusions from analysis protocol, the number of women included in analysis is 4,423 whites for GARNET, 5,202 white for WHIMS, 7,919 for SHARe African American and 3,377 for SHARe Hispanics.

Supplementary Note 2

STAGE 2 (FOCUSED FOLLOW-UP) STUDY DESCRIPTIONS

AA-DHS (African American Diabetes Heart Study): AA-DHS objectives are to improve understanding of ethnic differences in CAC and CP in populations of African and European ancestry. The AA-DHS consists of self-reported African Americans with T2D recruited from two Wake Forest School of Medicine (WFSM) studies: the family-based Diabetes Heart Study (DHS) and unrelated individuals in the AA-DHS. DHS is a cross-sectional study of European American and African American families with siblings concordant for T2D. AA-DHS started after DHS and enrolled unrelated African Americans. The AA-DHS GWAS utilized the Illumina 5M chip with imputation to 1,000 Genomes.

Airwave (The Airwave Health Monitoring Study): The Airwave Health Monitoring Study[19] was established to evaluate possible health risks associated with use of TETRA, a digital communication system used by police forces and other emergency services in Great Britain since 2001. The study has been broadened to investigate more generally the health of the work force. From 2004, participants from each force who agreed to participate were enrolled either with an enrolment questionnaire or a comprehensive health screening performed locally. This includes questionnaire, 7-day food diaries, anthropometry, measurements of cardiovascular and cognitive function, blood chemistry, coagulation and hematology. By March 2015, the study had recruited 53,606 participants, of whom 45,433 had attended the health screening, and 14,002 have genotype data (1000G imputed).

BES (Beijing Eye Study): The Beijing Eye Study is a population-based study that assesses the associated and risk factors of ocular and general diseases in a Chinese population. The study was initialized in 2001 and collected data from 4439 subjects aged $\geq$ 40 years and living in seven communities in the Beijing area. Three of these communities were located in a rural district and four were located in an urban district. The BES was followed-up in 2006, with 3251 of the original subjects participating, and in 2011, with 2695 subjects returning for the follow-up examination. At the examinations in 2006 and 2011, trained research staffs asked the subjects questions from a standard questionnaire providing information on the family status, level of education, income, quality of life, psychic depression, physical activity, and known major systemic diseases. Fasting blood samples were taken for measurement of concentrations of substances such as blood lipids, glucose, and glycosylated hemoglobin. Individuals were classified as self-reported non-smokers or self-reported current smokers. Alcohol consumption habits based on number of drinks per day were collected. Physical activity was assessed in questions on the number of hours per day and number of days per week spent on intensively or moderately performed sport activities, spent on walking, on riding a bicycle, and spent on sitting. All variables used in analyses were taken from examinations in 2006 or in 2011. The BES subjects were genotyped on two arrays, Illumina Human610-Quad (N = 832) and Illumina OmniExpress (N = 814).

BRIGHT (British Genetics of Hypertension): Participants of the BRIGHT Study are recruited from the Medical Research Council General Practice Framework and other primary care practices in the UK. Each case had a history of hypertension diagnosed prior to 60 years of age with confirmed blood

pressure recordings corresponding to seated levels >150/100mmHg (1 reading) or mean of 3 readings >145/95 mmHg. BRIGHT is focused on recruitment of hypertensive individuals with BMI<30. Sample selection for GWAS was based on DNA availability and quantity.[20]

CFS (Cleveland Family Study): The Cleveland Family Study (CFS) is a family-based, longitudinal study designed to characterize the genetic and non-genetic risk factors for sleep apnea. In total, 2534 individuals (46% African American) from 352 families were studied on up to 4 occasions over a period of 16 years (1990-2006). The initial aim of the study was to quantify the familial aggregation of sleep apnea. 632 African Americans were genotyped on the Affymetrix array 6.0 platform through the CARe Consortium with suitable genotying quality control. A further 122 African-Americans had genotyping based on the Illumina OmniExpress + Exome platform. Genomes were imputed separately for each chip based on a 1000 Genomes Project Phase 3 Version 5 cosmopolitan template using SHAPEIT and IMPUTE2.

Colaus (Cohorte Lausannoise): The cohort is a random population sample of the city of Lausanne aged 35-75 years. Recruitment began in June 2003 and ended in May 2006, and the first follow-up was conducted between April 2009 and September 2012. The CoLaus study was approved by the Institutional Ethics Committee of the University of Lausanne and informed consent was appropriately obtained by all participants. Both at baseline and follow-up, all participants attended the outpatient clinic of the University Hospital of Lausanne in the morning after an overnight fast. Data were collected by trained field interviewers in a single visit lasting about 60 min.

DESIR (Data from an Epidemiological Study on the Insulin Resistance): The DESIR cohort study aims to: describe and understand the relations between the abnormalities of the syndrome, their evolution, according to age and sex; search for risk factors of insulin resistance, in particular factors associated with the environment, lifestyle and genetic markers; quantify the links between the syndrome and both cardiovascular disease and diabetes; evaluate the frequency of the syndrome in terms of its consequences on public health.

DFTJ (Dongfeng-Tongji Cohort Study): The DFTJ-cohort study includes 27,009 retired employees from a state-owned automobile enterprise in China. This study was launched in 2008 and will be followed up every 5 years. In 2013 we conducted the first follow-up. By using semi-structural questionnaire and health examination, those having cancer or severe diseases were excluded. Fasting blood samples and detailed epidemiology data were collected. The main goal of the cohort was to identify the environmental and genetic risk factors and the gene-environment interactions on chronic diseases, and to find novel biomarkers for chronic disease and mortality prediction. Finally, 1,461 included in the present study with GWAS data. All of the participants wrote informed consent and the ethical committees in the Tongji Medical College approved this research project. Detailed information has been described in elsewhere[21].

QC criteria and imputation methods:
We did the GWAS scan on the DFTJ-cohort with Affymetrix Genome-Wide Human SNP Array 6.0 chips. In total, we genotyped 906,703 SNPs among 1,461 subjects. After stringent QC filtering, SNPs with MAF < 0.01, Hardy-Weinberg Equilibrium (HWE) < 0.0001, and SNP call rate < 95% were excluded. Individuals with call rates < 95% were also not included for further analysis. In total, we

retained 1,452 subjects with 658,288 autosomal SNPs for statistical analyses, with an overall call rate of 99.68%. We used MACH 1.0 software to impute untyped SNPs using the LD information from the HapMap phase II database (CHB+JPT as a reference set (2007-08_rel22, released 2007-03-02). Imputed SNPs with high genotype information content (Rsq > 0.3 for MACH) were kept for the further association analysis.

DHS (Diabetes Heart Study): The Diabetes Heart Study (DHS) is an ongoing family-based cohort study investigating the epidemiology and genetics of cardiovascular disease (CVD) in a population-based sample. The DHS recruited T2D-affected siblings without advanced renal insufficiency from 1998 through 2005 in western North Carolina. DHS has collected genetic data on 1,220 self-described European American (EA) individuals from 475 families. Genotyping was completed using an Affymetrix Genome-Wide Human SNP Array 5.0 with imputation of 1,000 Genomes project SNPs from this array using IMPUTE2 and the Phase I v2, cosmopolitan (integrated) reference panel, build 37.

DR's EXTRA (Dose-Responses to Exercise Training): The Dose-Responses to Exercise Training (DR's EXTRA) Study is a 4-year RCT on the effects of regular physical exercise and healthy diet on endothelial function, atherosclerosis and cognition in a randomly selected population sample (n=3,000) of Eastern Finnish men and women, identified from the national population register, aged 55-74 years. Of the eligible sample, 1,410 individuals were randomized into one of the 6 groups: aerobic exercise, resistance exercise, diet, combined aerobic exercise and diet, combined resistance exercise and diet, or reference group following baseline assessments. During the four year intervention the drop-out rate was 15%.

EGCUT (Estonian Genome Center - University of Tartu (Estonian Biobank)): The Estonian Biobank is the population-based biobank of the Estonian Genome Center at the University of Tartu (www.biobank.ee; EGCUT). The entire project is conducted according to the Estonian Gene Research Act and all of the participants have signed the broad informed consent. The cohort size is up to 51,535 individuals from 18 years of age and up, which closely reflects the age, sex and geographical distribution of the Estonian population. All of the subjects are recruited randomly by general practitioners and physicians in hospitals. A Computer Assisted Personal interview is filled within 1-2 hours at a doctor's office, which includes personal, genealogical, educational, occupational history and lifestyle data. Anthropometric measurements, blood pressure and resting heart rate are measured and venous blood taken during the visit. Medical history and current health status is recorded according to ICD-10 codes.

EPIC (European Prospective Investigation into Cancer and Nutrition)-Norfolk: The European Prospective Investigation of Cancer (EPIC) began as a large multi-centre cohort study primarily looking at the connection between diet, lifestyle factors and cancer, although the study was broadened from the outset to include other conditions. The EPIC-Norfolk participants are men and women who were aged between 40 and 79 when they joined the study and who lived in Norwich and the surrounding towns and rural areas. They have been contributing information about their diet, lifestyle and health through questionnaires and health checks over two decades. The Norwich Local Research Ethics Committee granted ethical approval for the study. All participants gave written informed consent.

FUSION (Finland-United States Investigation of NIDDM Genetics): The Finland-United States Investigation of NIDDM Genetics (FUSION) study is a long-term effort to identify genetic variants that predispose to type 2 diabetes (T2D) or that impact the variability of T2D-related quantitative traits. The FUSION GWAS sample consists of 1,161 Finnish T2D cases and 1,174 Finnish normal glucose-tolerant (NGT) controls[22]. Cases are defined by fasting plasma glucose ≥ 7.0 mmol/l or 2-h plasma glucose ≥ 11.1 mmol/l, by report of diabetes medication use, or based on medical record review. 789 FUSION cases each reported at least one T2D sibling; 372 Finrisk 2002 T2D cases came from a Finnish population-based risk factor survey. NGT controls are defined by fasting glucose < 6.1 mmol/l and 2-h glucose < 7.8 mmol/l. FUSION controls include 119 subjects from Vantaa, Finland who were NGT at ages 65 and 70 years, 304 NGT spouses from FUSION families, and 651 Finrisk 2002 subjects. The controls were approximately frequency matched to the cases by age, sex, and birth province. Smoking and alcohol data are only available in the FUSION subset of our GWAS samples.

GeneSTAR (Genetic Studies of Atherosclerosis Risk): GeneSTAR is a family-based prospective study of more than 4000 participants begun in 1983 to determine phenotypic and genetic causes of premature cardiovascular disease. Families were identified from 1983-2006 from probands with a premature coronary disease event prior to 60 years of age who were identified at the time of hospitalization in any of 10 hospitals in the Baltimore, Maryland area. Their apparently healthy 30-59 year old siblings without known coronary disease were recruited and screened between 1983 and 2006. From 2003-2006, adult offspring over 21 years of age of all participating siblings and probands, as well as the coparents of the offspring were recruited and screened. Genotyping was performed in 3,232 participants on the Illumina 1Mv1_c platform.

GLACIER (Gene x Lifestyle Interactions and Complex Traits Involved in Elevated Disease Risk): The Gene-Lifestyle interactions And Complex traits Involved in Elevated disease Risk (GLACIER) Study[23] is nested within the Västerbotten Intervention Programme, which is part of the Northern Sweden Health and Disease Study, a population-based prospective cohort study from northern Sweden. Participants were genotyped with Illumina CardioMetaboChip array. This array contains ~200,000 variants, the majority being common variants. Analysis of serum lipids (HDL-C, triglycerides and total cholesterol) were undertaken at the Department of Clinical Chemistry at Umeå University Hospital using routine methods. LDL-C was determined using the Friedewald formula. All participants completed a detailed, optically readable, health and lifestyle questionnaire including questions about smoking status and alcohol intake (FFQ).

GRAPHIC (Genetic Regulation of Arterial Pressure of Humans in the Community): The GRAPHIC Study comprises 2024 individuals from 520 nuclear families recruited from the general population in Leicestershire, UK between 2003-2005 for the purpose of investigating the genetic determinants of blood pressure and related cardiovascular traits. A detailed medical history was obtained from study subjects by standardized questionnaires and clinical examination was performed by research nurses following standard procedures. Measurements obtained included height, weight, waist-hip ratio, clinic and ambulatory blood pressure and a 12-lead ECG.

HCHS/SOL (Hispanic Community Health Study/ Study of Latinos): The HCHS/SOL is a community-based cohort study of 16,415 self-identified Hispanic/Latino persons aged 18–74 years and selected from households in predefined census-block groups across four US field centers (in Chicago,

Miami, the Bronx, and San Diego). The census-block groups were chosen to provide diversity among cohort participants with regard to socioeconomic status and national origin or background. The HCHS/SOL cohort includes participants who self-identified as having a Hispanic/Latino background; the largest groups are Central American (n = 1,730), Cuban (n = 2,348), Dominican (n = 1,460), Mexican (n = 6,471), Puerto Rican (n = 2,728), and South American (n = 1,068). The HCHS/SOL baseline clinical examination occurred between 2008 and 2011 and included comprehensive biological, behavioral, and sociodemographic assessments. Consenting HCHS/SOL subjects were genotyped at Illumina on the HCHS/SOL custom 15041502 B3 array. The custom array comprised the Illumina Omni 2.5M array (HumanOmni2.5-8v.1-1) ancestry-informative markers, known GWAS hits and drug absorption, distribution, metabolism, and excretion (ADME) markers, and additional custom content including ~150,000 SNPs selected from the CLM (Colombian in Medellin, Colombia), MXL (Mexican Ancestry in Los Angeles, California), and PUR (Puerto Rican in Puerto Rico) samples in the 1000Genomes phase 1 data to capture a greater amount of Amerindian genetic variation. QA/QC procedures yielded a total of 12,803 unique study participants for imputation and downstream association analyses.

HRS (Health and Retirement Study): The Health and Retirement Study (HRS) is a longitudinal survey of a representative sample of Americans over the age of 50[24-25]. The current sample is over 26,000 persons in 17,000 households. Respondents are interviewed every two years about income and wealth, health and use of health services, work and retirement, and family connections. DNA was extracted from saliva collected during a face-to-face interview in the respondents' homes. These data represent respondents who provided DNA samples and signed consent forms in 2006, 2008, and 2010. Respondents were removed if they had missing genotype or phenotype data.

HyperGEN-AXIOM (Hypertension Genetic Epidemiology Network): HyperGEN is a family-based study that investigates the genetic causes of hypertension and related conditions in EA and AA subjects. HyperGEN recruited hypertensive sibships, along with their normotensive adult offspring, and an age-matched random sample. HyperGEN has collected data on 2,471 Caucasian-American subjects and 2,300 African-American subjects, from five field centers in Alabama, Massachusetts, Minnesota, North Carolina, and Utah. HyperGEN participates as a discovery study using GWAS available in a large subset of the samples. The remaining AA subjects without GWAS data were genotyped on the Affymetrix Axiom chip as part of a HyperGEN admixture mapping ancillary study. After excluding subjects already included in the original HyperGEN (or with family members included), this subset of approximately 450 AA subjects are included in the HyperGEN-AXIOM study which participates in replications.

INGI-CARL and INGI-FVG (Italian Network Genetic Isolates): INGI-FVG and INGI-CARL studies include samples coming from isolated populations and belong to the ITALIAN NETWORK OF GENETIC ISOLATES (INGI). INGI-CARL examined about 1000 subjects between 1998 and 2005 coming from a small village of the South of Italy situated in the extreme northern part of Puglia Region, while INGI FVG involved about 1700 subjects between 2008 and 2011 coming from six different villages located in the North-East of Italy in Friuli Venezia Giulia region. A questionnaire was administered to each participant to obtain socio-demographic information, as well as data on professional activity, family history, eating habits and lifestyle, such as smoking, coffee and alcohol consumption, physical activity. Furthermore, a medical screening, including anamnesis, blood pressure,

drugs and clinical chemistry evaluation (blood count and different biochemical parameters, such as lipids) were made. All participants gave their written informed consent.

IRAS Family Study (Insulin Resistance Atherosclerosis Study): The IRASFS was a family study designed to examine the genetic and epidemiologic basis of glucose homeostasis traits and abdominal adiposity. Briefly, self-reported Mexican pedigrees were recruited in San Antonio, TX and San Luis Valley, CO. Probands with large families were recruited from the initial non-family-based IRAS, which was modestly enriched for impaired glucose tolerance and T2D. Inclusion of IRASFS data is limited to 1040 normoglycemic individuals in 88 pedigrees with genotype data from the Illumina OmniExpress and Omni 1S arrays and imputation to the 1000 Genome Integrated Reference Panel (phase I).

JUPITER (Justification for the Use of Statins in Primary Prevention: An Intervention Trial Evaluating Rosuvastatin): Genetic analysis was performed in a sub-population from JUPITER (Justification for the Use of statins in Prevention: an Intervention Trial Evaluating Rosuvastatin), an international, randomized, placebo-controlled trial of rosuvastatin (20mg/day) in the primary prevention of cardiovascular disease conducted among apparently healthy men and women with LDL-C < 130 mg/dL and hsCRP ≥ 2 mg/L[26,27]. Individuals with diabetes or triglyceride concentration >500mg/dL were excluded. The present analysis includes only individuals who provided consent for genetic analysis, had successfully collected genotype information, and who had either verified European or verified South African black ancestry.

KORA (Cooperative Health Research in the Augsburg Region): The KORA study is a series of independent population-based epidemiological surveys of participants living in the region of Augsburg, Southern Germany. All survey participants are residents of German nationality identified through the registration office and were examined in 1994/95 (KORA S3) and 1999/2001 (KORA S4). In the KORA S3 and S4 studies 4,856 and 4,261 subjects have been examined implying response rates of 75% and 67%, respectively. 3,006 subjects participated in a 10-year follow-up examination of S3 in 2004/05 (KORA F3), and 3080 of S4 in 2006/2008 (KORA F4). The age range of the participants was 25 to 74 years at recruitment. Informed consent has been given by all participants. The study has been approved by the local ethics committee. Individuals for genotyping in KORA F3 and KORA F4 were randomly selected and these genotypes are taken for the analysis of the phenotypes in KORA S3 and KORA S4.

LBC1936 (Lothian Birth Cohort 1936): LBC1936 consists of 1091 (548 male) relatively healthy individuals who underwent cognitive and medical testing at a mean age of 69.6 years (SD = 0.8). They were born in 1936, most took part in the Scottish Mental Survey of 1947, and almost all lived independently in the Lothian region of Scotland.[28]

Lifelines (Netherlands Biobank): Lifelines (https://lifelines.nl/) is a multi-disciplinary prospective population-based cohort study using a unique three-generation design to examine the health and health-related behaviors of 165,000 persons living in the North East region of The Netherlands[29]. It employs a broad range of investigative procedures in assessing the biomedical, socio-demographic, behavioral, physical and psychological factors which contribute to the health and disease of the general population, with a special focus on multimorbidity. In addition, the Lifelines project comprises a number of cross-sectional sub-studies which investigate specific age-related conditions. These include investigations

into metabolic and hormonal diseases, including obesity, cardiovascular and renal diseases, pulmonary diseases and allergy, cognitive function and depression, and musculoskeletal conditions. All survey participants are between 18 and 90 years old at the time of enrollment. Recruitment has been going on since the end of 2006, and over 130,000 participants had been included by April 2013. At the baseline examination, the participants in the study were asked to fill in a questionnaire (on paper or online) before the first visit. During the first and second visit, the first or second part of the questionnaire, respectively, are checked for completeness, a number of investigations are conducted, and blood and urine samples are taken. Lifelines is a facility that is open for all researchers. Information on application and data access procedure is summarized on www.lifelines.nl.

METSIM (Metabolic Syndrome In Men): The METSIM Study includes 10,197 men, aged from 45 to 73 years at recruitment, randomly selected from the population register of the Kuopio town, Eastern Finland, and examined in 2005-2010[30]. The aim of the study is to investigate genetic and non-genetic factors associated with type 2 diabetes and cardiovascular disease and its risk factors.

NESDA (Netherlands Study of Depression and Anxiety): NESDA is a multi-center study designed to examine the long-term course and consequences of depressive and anxiety disorders (http://www.nesda.nl)[31]. NESDA included both individuals with depressive and/or anxiety disorders and controls without psychiatric conditions. Inclusion criteria were age 18-65 years and self-reported western European ancestry while exclusion criteria were not being fluent in Dutch and having a primary diagnosis of another psychiatric condition (psychotic disorder, obsessive compulsive disorder, bipolar disorder, or severe substance use disorder).

PREVEND(The Prevention of REnal and Vascular ENd stage Disease study): The PREVEND study is an ongoing prospective study investigating the natural course of increased levels of urinary albumin excretion and its relation to renal and cardiovascular disease. Inhabitants 28 to 75 years of age (n=85,421) in the city of Groningen, The Netherlands, were asked to complete a short questionnaire, 47% responded, and individuals were then selected with a urinary albumin concentration of at least 10 mg/L (n = 7,768) and a randomly selected control group with a urinary albumin concentration less than 10 mg/L (n = 3,395). Details of the protocol have been described elsewhere[32].

RHS (Ragama Health Study): The Ragama Health Study (RHS) is a population-based study of South Asian men and women aged 35-64yrs living in the Ragama Medical Officer of Health (MOH) area, near Colombo, Sri Lanka.[33] Consenting adults attended a clinic after a 12-h fast with available health records, and were interviewed by trained personnel to obtain information on medical, sociodemographic, and lifestyle variables. A 10-mL sample of venous blood was obtained from each subject. The concurrent study was performed in two tea plantation estates in the Lindula MOH area, near Nuwara Eliya (180 km from Colombo), to investigate the gene-environment interaction in a community with differing lifestyles (e.g., physical activity and diet). The RHS is a collaborative effort between the Faculty of Medicine, University of Kelaniya and the National Center for Global Health and Medicine, Japan.

SHEEP (Stockholm Heart Epidemiology Project): The SHEEP is a population based case-control study of risk factors for first episode of acute myocardial infarction. The study base comprised all

Swedish citizens resident in the Stockholm county 1992-1994 who were 45-70 years of age and were free of previous clinically diagnosed myocardial infarction.

Cases were identified using three different sources: 1) coronary units and internal medicine wards for acute care in all Stockholm hospitals; 2) the National Patient Register; and 3) death certificates. For the present study, only cases who survived at least 28 days were considered (n=1213).

First time incident myocardial infarction cases (n=1213) were identified during a 2-year period (1992-1993) for men and during a 3-year period (1992-1994) for women. Controls (n=1561) were randomly recruited from the study population continuously over time within 2 days of the case occurrence and matched to cases on age (5-years interval), sex and hospital catchment area using computerized registers of the population of Stockholm. Five control candidates were sampled simultaneously to be able to replace potential non-respondent controls. Occasionally, because of late response of the initial control, both the first and alternative controls were considered resulting in the inclusion of more controls than cases. Postal questionnaires covering a wide range of exposure areas including occupational exposures, life style factors, social factors and health related factors were distributed to the participants. Clinical investigations were performed at least three months after myocardial infarction of cases and their matched controls. The investigations included blood samplings under fasting conditions with collection of whole blood for DNA extraction, serum and plasma. A biobank was established containing DNA, serum and plasma.

Exposure information based on both the questionnaire and biological data from the health examination was available for 78% of the male and 67% of the female non-fatal cases; the corresponding figures for their controls were 68% and 64%.

SWHS/SMHS (Shanghai Women's Health Study/ Shanghai Men's Health Study): The Shanghai Women's Health Study (SWHS) is an ongoing population-based cohort study of approximately 75,000 women who were aged 40-70 years at study enrollment and resided in in urban Shanghai, China; 56,832 (75.8%) provided a blood samples. Recruitment for the SWHS was initiated in 1997 and completed in 2000. The self-administered questionnaire includes information on demographic characteristics, disease and surgery histories, personal habits (such as cigarette smoking, alcohol consumption, tea drinking, and ginseng use), menstrual history, residential history, occupational history, and family history of cancer. Included in the current project were all women who had GWAS data and lipid measurements at the baseline interview.

The Shanghai Men’s Health Study (SMHS) is an ongoing population-based cohort study of 61,480 Chinese men who were aged between 40 and 74 years, were free of cancer at enrollment, and lived in urban Shanghai, China; 45,766 (74.4%) provided a blood samples. Recruitment for the SMHS was initiated in 2002 and completed in 2006. The self-administered questionnaire includes information on demographic characteristics, disease and surgery histories, personal habits (such as cigarette smoking, alcohol consumption, tea drinking, and ginseng use), residential history, occupational history, and family history of cancer. Included in the current project were 298 men who had GWAS data and lipid measurements at the baseline interview.

Genotyping and imputation: Genomic DNA was extracted from buffy coats by using a Qiagen DNA purification kit (Valencia, CA) or Puregene DNA purification kit (Minneapolis, MN) according to the manufacturers' instructions and then used for genotyping assays. The GWAS genotyping was performed using the Affymetrix Genome-Wide Human SNP Array 6.0 (Affy6.0) platform or Illumina 660, following manufacturers' protocols. After sample quality control, we exclude SNPs with 1) MAF <0.01; 2) call rate <95%; 2) bad genotyping cluster; and 3) concordance rate <95% among duplicated QC samples. Genotypes were imputed using the program MACH (http://www.sph.umich.edu/csg/abecasis/MACH/download/), which determines the probable distribution of missing genotypes conditional on a set of known haplotypes, while simultaneously estimating the fine-scale recombination map. Phased autosome SNP data from HapMap Phase II Asians (release 22) were used as the reference. To test for associations between the imputed SNP data with BMI, linear regression (additive model) was used, in which SNPs were represented by the expected allele count, an approach that takes into account the degree of uncertainty of genotype imputation (http://www.sph.umich.edu/csg/abecasis/MACH/download/).

The lipid profiles were measured at Vanderbilt Lipid Laboratory. Total cholesterol, high-density lipoprotein (HDL) cholesterol, and triglycerides (TG) were measured using an ACE Clinical Chemistry System (Alfa Wassermann, Inc, West Caldwell, NJ). Low-density lipoprotein (LDL) cholesterol levels were calculated by using the Friedewald equation. The levels of LDL cholesterol were directly measured using an ACE Clinical Chemistry System for subjects with TG levels ≥ 400 mg/dL. Fasting status was defined as an interval between the last meal and blood draw of 8 hours or longer.

TRAILS (Tracking Adolescents' Individual Lives Survey): TRAILS is a prospective cohort study of Dutch adolescents and young adults, with bi- or triennial measurements from age 11 onwards, which started in 2001. TRAILS consists of a general population and a clinical cohort (https://www.trails.nl/en/home). In the population cohort, six assessment waves have been completed to date, at mean ages 11.1 (SD = 0.6), 13.6 (SD = 0.5), 16.3 (SD = 0.7), 19.1 (SD = 0.6), 22.3 (SD = 0.6), and 25.8 (SD = 0.6). Data for the present study were collected in the population cohort only, during the third assessment wave. The study was approved by the Dutch Central Committee on Research Involving Human Subjects.

TWINGENE (TwinGene of the Swedish Twin Registry): The aim of the TwinGene project has been to systematically transform the oldest cohorts of the Swedish Twin Registry (STR) into a molecular-genetic resource. Beginning in 2004, about 200 twins were contacted each month until the data collection was completed in 2008. A total of 21 500 twins were contacted where of 12 600 participated. Invitations to the study contained information of the study and its purpose. Along with the invitations consent forms and health questionnaire were sent to the subjects. When the signed consent forms where returned, the subjects were sent blood sampling equipment and asked to contact a local health facility for blood sampling. The study population was recruited among twins participating in the Screening Across the Lifespan Twin Study (SALT) which was a telephone interview study conducted in 1998-2002. Other inclusion criteria were that both twins in the pair had to be alive and living in Sweden. Subjects were excluded from the study if they preciously declined participation in future studies or if they had been enrolled in other STR DNA sampling projects. The subjects were asked to make an appointment for a health check-up at their local health-care facility on the morning Monday to Thursday and not the day before a national holiday, this to ensure that the sample would reach the KI

biobank the following morning by overnight mail. The subjects were instructed to fast from 20.00 the previous night. By venipuncture a total of 50 ml of blood was drawn from each subject. Tubes with serum and blood for biobanking as well as for clinical chemistry tests were sent to KI by overnight mail. One 7ml EDTA tube of whole blood is stored in -80°C while a second 7ml EDTA tube of blood is used for DNA extraction using Puregene extraction kit (Gentra systems, Minneapolis, USA). After excluding subjects in which the DNA concentration in the stock-solution was below 20ng/µl as well as subset of 302 female monozygous twin pairs participating in a previous genome wide effort DNA from 9896 individual subjects was sent to SNP&SEQ Technology Platform Uppsala, Sweden for genome wide genotyping with Illumina OmniExpress bead chip (all available dizygous twins + one twin from each available MZ twin pair).

YFS (The Cardiovascular Risk in Young Finns Study): The YFS is a population-based follow up-study started in 1980. The main aim of the YFS is to determine the contribution made by childhood lifestyle, biological and psychological measures to the risk of cardiovascular diseases in adulthood. In 1980, over 3,500 children and adolescents all around Finland participated in the baseline study. The follow-up studies have been conducted mainly with 3-year intervals. The latest 30-year follow-up study was conducted in 2010-11 (ages 33-49 years) with 2,063 participants. The study was approved by the local ethics committees (University Hospitals of Helsinki, Turku, Tampere, Kuopio and Oulu) and was conducted following the guidelines of the Declaration of Helsinki. All participants gave their written informed consent.

Supplementary Note 3

STAGE 1 STUDY ACKNOWLEDGMENTS:

Infrastructure for the CHARGE Consortium is supported in part by the National Heart, Lung, and Blood Institute grant R01HL105756. Infrastructure for the Gene-Lifestyle Working Group is supported by the National Heart, Lung, and Blood Institute grant R01HL118305. Tuomas O. Kilpeläinen was supported in part by the Danish Council for Independent Research (DFF–1333-00124 and DFF–1331-00730B) and the Novo Nordisk Foundation (NNF18CC0034900, NNF17OC0026848 and NNF15CC0018486).

AGES (Age Gene/Environment Susceptibility Reykjavik Study): This study has been funded by NIH contract N01-AG012100, the NIA Intramural Research Program, an Intramural Research Program Award (ZIAEY000401) from the National Eye Institute, an award from the National Institute on Deafness and Other Communication Disorders (NIDCD) Division of Scientific Programs (IAA Y2-DC_1004-02), Hjartavernd (the Icelandic Heart Association), and the Althingi (the Icelandic Parliament). The study is approved by the Icelandic National Bioethics Committee, VSN: 00-063. The researchers are indebted to the participants for their willingness to participate in the study.

ARIC (Atherosclerosis Risk in Communities) Study: The ARIC study is carried out as a collaborative study supported by National Heart, Lung, and Blood Institute contracts (HHSN268201100005C, HHSN268201100006C, HHSN268201100007C, HHSN268201100008C, HHSN268201100009C, HHSN268201100010C, HHSN268201100011C, and HHSN268201100012C), R01HL087641, R01HL59367 and R01HL086694; National Human Genome Research Institute contract U01HG004402; and National Institutes of Health contract HHSN268200625226C. The authors thank the staff and participants of the ARIC study for their important contributions. Infrastructure was partly supported by Grant Number UL1RR025005, a component of the National Institutes of Health and NIH Roadmap for Medical Research.

Baependi Heart Study (Brazil): The Baependi Heart Study was supported by Fundação de Amparo a Pesquisa do Estado de São Paulo (FAPESP) (Grant 2013/17368-0), Coordenação de Aperfeiçoamento de Pessoal de Nível Superior (CAPES) and Hospital Samaritano Society (Grant 25000.180.664/2011-35), through Ministry of Health to Support Program Institutional Development of the Unified Health System (SUS-PROADI).

CARDIA (Coronary Artery Risk Development in Young Adults): The CARDIA Study is conducted and supported by the National Heart, Lung, and Blood Institute in collaboration with the University of Alabama at Birmingham (HHSN268201300025C & HHSN268201300026C), Northwestern University (HHSN268201300027C), University of Minnesota (HHSN268201300028C), Kaiser Foundation Research Institute (HHSN268201300029C), and Johns Hopkins University School of Medicine (HHSN268200900041C). CARDIA is also partially supported by the Intramural Research Program of the National Institute on Aging. Genotyping was funded as part of the NHLBI Candidate-gene Association Resource (N01-HC-65226) and the NHGRI Gene Environment Association Studies

(GENEVA) (U01-HG004729, U01-HG04424, and U01-HG004446). This manuscript has been reviewed and approved by CARDIA for scientific content.

CHS (Cardiovascular Health Study): This CHS research was supported by NHLBI contracts HHSN268201200036C, HHSN268200800007C, HHSN268200960009C, HHSN268201800001C, N01HC55222, N01HC85079, N01HC85080, N01HC85081, N01HC85082, N01HC85083, N01HC85086; and NHLBI grants U01HL080295, R01HL085251, R01HL087652, R01HL105756, R01HL103612, R01HL120393 and R01HL130114 with additional contribution from the National Institute of Neurological Disorders and Stroke (NINDS). Additional support was provided through R01AG023629 from the National Institute on Aging (NIA). A full list of principal CHS investigators and institutions can be found at CHS-NHLBI.org. The provision of genotyping data was supported in part by the National Center for Advancing Translational Sciences, CTSI grant UL1TR001881, and the National Institute of Diabetes and Digestive and Kidney Disease Diabetes Research Center (DRC) grant DK063491 to the Southern California Diabetes Endocrinology Research Center. The content is solely the responsibility of the authors and does not necessarily represent the official views of the National Institutes of Health.

CROATIA-Korcula: We would like to acknowledge the staff of several institutions in Croatia that supported the field work, including but not limited to The University of Split and Zagreb Medical Schools and the Croatian Institute for Public Health. We would like to acknowledge the invaluable contributions of the recruitment team in Korcula, the administrative teams in Croatia and Edinburgh and the participants. The SNP genotyping for the CROATIA-Korcula cohort was performed in Helmholtz Zentrum München, Neuherberg, Germany. CROATIA-Korcula (CR-Korcula) was funded by the Medical Research Council UK, The Croatian Ministry of Science, Education and Sports (grant 216-1080315-0302), the European Union framework program 6 EUROSPAN project (contract no. LSHG-CT-2006-018947), the Croatian Science Foundation (grant 8875) and the Centre of Competencies for Integrative Treatment, Prevention and Rehabilitation using TMS.

CROATIA-Vis: We would like to acknowledge the staff of several institutions in Croatia that supported the field work, including but not limited to The University of Split and Zagreb Medical Schools, the Institute for Anthropological Research in Zagreb and Croatian Institute for Public Health. The SNP genotyping for the CROATIA-Vis cohort was performed in the core genotyping laboratory of the Wellcome Trust Clinical Research Facility at the Western General Hospital, Edinburgh, Scotland. CROATIA-Vis (CR-Vis) was funded by the Medical Research Council UK, The Croatian Ministry of Science, Education and Sports (grant 216-1080315-0302), and the European Union framework program 6 EUROSPAN project (contract no. LSHG-CT-2006-018947).

ERF (Erasmus Rucphen Family study): The ERF study as a part of EUROSPAN (European Special Populations Research Network) was supported by European Commission FP6 STRP grant number 018947 (LSHG-CT-2006-01947) and also received funding from the European Community's Seventh Framework Programme (FP7/2007-2013)/grant agreement HEALTH-F4-2007-201413 by the European Commission under the programme "Quality of Life and Management of the Living Resources" of 5th Framework Programme (no. QLG2-CT-2002-01254). The ERF study was further supported by ENGAGE consortium and CMSB. High-throughput analysis of the ERF data was supported by joint grant from Netherlands Organisation for Scientific Research and the Russian Foundation for Basic

Research (NWO-RFBR 047.017.043). ERF was further supported by the ZonMw grant (project 91111025). We are grateful to all study participants and their relatives, general practitioners and neurologists for their contributions and to P. Veraart for her help in genealogy, J. Vergeer for the supervision of the laboratory work, P. Snijders for his help in data collection and E.M. van Leeuwen for genetic imputation.

FamHS (Family Heart Study): The FamHS is funded by R01HL118305 and R01HL117078 NHLBI grants, and 5R01DK07568102 and 5R01DK089256 NIDDK grant.

FHS (Framingham Heart Study): This research was conducted in part using data and resources from the Framingham Heart Study of the National Heart Lung and Blood Institute of the National Institutes of Health and Boston University School of Medicine. The analyses reflect intellectual input and resource development from the Framingham Heart Study investigators participating in the SNP Health Association Resource (SHARe) project. This work was partially supported by the National Heart, Lung and Blood Institute's Framingham Heart Study (Contract Nos. N01-HC-25195 and HHSN268201500001I) and its contract with Affymetrix, Inc for genotyping services (Contract No. N02-HL-6-4278). A portion of this research utilized the Linux Cluster for Genetic Analysis (LinGA-II) funded by the Robert Dawson Evans Endowment of the Department of Medicine at Boston University School of Medicine and Boston Medical Center. This research was partially supported by grant R01-DK089256 from the National Institute of Diabetes and Digestive and Kidney Diseases (MPIs: Ingrid B. Borecki, L. Adrienne Cupples, Kari North).

GENOA (Genetic Epidemiology Network of Arteriopathy): Support for GENOA was provided by the National Heart, Lung and Blood Institute (HL119443, HL118305, HL054464, HL054457, HL054481, HL071917 and HL087660) of the National Institutes of Health. Genotyping was performed at the Mayo Clinic (Stephen T. Turner, MD, Mariza de Andrade PhD, Julie Cunningham, PhD). We thank Eric Boerwinkle, PhD and Megan L. Grove from the Human Genetics Center and Institute of Molecular Medicine and Division of Epidemiology, University of Texas Health Science Center, Houston, Texas, USA for their help with genotyping. We would also like to thank the families that participated in the GENOA study.

GenSalt (Genetic Epidemiology Network of Salt Sensitivity): The Genetic Epidemiology Network of Salt Sensitivity is supported by research grants (U01HL072507, R01HL087263, and R01HL090682) from the National Heart, Lung, and Blood Institute, National Institutes of Health, Bethesda, MD.

GOLDN (Genetics of Diet and Lipid Lowering Network): Support for the genome-wide association studies in GOLDN was provided by the National Heart, Lung, and Blood Institute grant U01HL072524-04 and R01HL091357.

GS:SFHS: Generation Scotland received core support from the Chief Scientist Office of the Scottish Government Health Directorates [CZD/16/6] and the Scottish Funding Council [HR03006]. Genotyping of the GS:SFHS samples was carried out by the Genetics Core Laboratory at the Wellcome Trust Clinical Research Facility, Edinburgh, Scotland and was funded by the Medical Research Council UK and the Wellcome Trust (Wellcome Trust Strategic Award “STratifying Resilience and Depression Longitudinally” (STRADL) Reference 104036/Z/14/Z). Ethics approval for the study was

given by the NHS Tayside committee on research ethics (reference 05/S1401/89). We are grateful to all the families who took part, the general practitioners and the Scottish School of Primary Care for their help in recruiting them, and the whole Generation Scotland team, which includes interviewers, computer and laboratory technicians, clerical workers, research scientists, volunteers, managers, receptionists, healthcare assistants and nurses.

HANDLS (Healthy Aging in Neighborhoods of Diversity across the Life Span): The Healthy Aging in Neighborhoods of Diversity across the Life Span (HANDLS) study was supported by the Intramural Research Program of the NIH, National Institute on Aging and the National Center on Minority Health and Health Disparities (project # Z01-AG000513 and human subjects protocol number 09-AG-N248). Data analyses for the HANDLS study utilized the high-performance computational resources of the Biowulf Linux cluster at the National Institutes of Health, Bethesda, MD. (http://biowulf.nih.gov; http://hpc.nih.gov).

Health ABC (Health, Aging, and Body Composition): Health ABC was funded by the National Institutes of Aging. This research was supported by NIA contracts N01AG62101, N01AG62103, and N01AG62106. The GWAS was funded by NIA grant 1R01AG032098-01A1 to Wake Forest University Health Sciences and genotyping services were provided by the Center for Inherited Disease Research (CIDR). CIDR is fully funded through a federal contract from the National Institutes of Health to The Johns Hopkins University, contract number HHSN268200782096C. This research was supported in part by the Intramural Research Program of the NIH, National Institute on Aging.

HUFS (Howard University Family Study): The Howard University Family Study was supported by National Institutes of Health grants S06GM008016-320107 to Charles Rotimi and S06GM008016-380111 to Adebowale Adeyemo. We thank the participants of the study, for which enrollment was carried out at the Howard University General Clinical Research Center, supported by National Institutes of Health grant 2M01RR010284. The contents of this publication are solely the responsibility of the authors and do not necessarily represent the official view of the National Institutes of Health. This research was supported in part by the Intramural Research Program of the Center for Research on Genomics and Global Health (CRGGH). The CRGGH is supported by the National Human Genome Research Institute, the National Institute of Diabetes and Digestive and Kidney Diseases, the Center for Information Technology, and the Office of the Director at the National Institutes of Health (Z01HG200362). Genotyping support was provided by the Coriell Institute for Medical Research.

HyperGEN (Hypertension Genetic Epidemiology Network): The Hypertension Network is funded by cooperative agreements (U10) with NHLBI: HL54471, HL54472, HL54473, HL54495, HL54496, HL54497, HL54509, HL54515, and 2 R01 HL55673-12. The study involves: University of Utah: (Network Coordinating Center, Field Center, and Molecular Genetics Lab); Univ. of Alabama at Birmingham: (Field Center and Echo Coordinating and Analysis Center); Medical College of Wisconsin: (Echo Genotyping Lab); Boston University: (Field Center); University of Minnesota: (Field Center and Biochemistry Lab); University of North Carolina: (Field Center); Washington University: (Data Coordinating Center); Weil Cornell Medical College: (Echo Reading Center); National Heart, Lung, & Blood Institute. For a complete list of HyperGEN Investigators: http://www.biostat.wustl.edu/hypergen/Acknowledge.html

JHS (Jackson Heart Study): The Jackson Heart Study is supported by contracts HSN268201300046C, HHSN268201300047C, HHSN268201300048C, HHSN268201300049C, HHSN268201300050C from the National Heart, Lung, and Blood Institute on Minority Health and Health Disparities. The authors acknowledge the Jackson Heart Study team institutions (University of Mississippi Medical Center, Jackson State University and Tougaloo College) and participants for their long-term commitment that continues to improve our understanding of the genetic epidemiology of cardiovascular and other chronic diseases among African Americans.

MESA (Multi-Ethnic Study of Atherosclerosis: This research was supported by the Multi-Ethnic Study of Atherosclerosis (MESA) contracts HHSN268201500003I, N01-HC-95159, N01-HC-95160, N01-HC-95161, N01-HC-95162, N01-HC-95163, N01-HC-95164, N01-HC-95165, N01-HC-95166, N01-HC-95167, N01-HC-95168, N01-HC-95169, UL1-TR-000040, UL1-TR-001079, and UL1-TR-001420. Funding for MESA Share genotyping was provided by NHLBI Contract N02-HL-6-4278. This publication was partially developed under a STAR research assistance agreement, No. RD831697 (MESA Air), awarded by the U.S Environmental Protection Agency. It has not been formally reviewed by the EPA. The views expressed in this document are solely those of the authors and the EPA does not endorse any products or commercial services mentioned in this publication. The provision of genotyping data was supported in part by the National Center for Advancing Translational Sciences, CTSI grant UL1TRO1881, and the National Institute of Diabetes and Digestive and Kidney Disease Diabetes Research Center (DRC) grant DK063491 to the Southern California Diabetes Endocrinology Research Center. The authors thank the participants of the MESA study, the Coordinating Center, MESA investigators, and study staff for their valuable contributions. A full list of participating MESA investigators and institutions can be found at http://www.mesa-nhlbi.org.

NEO (The Netherlands Epidemiology of Obesity study): The authors of the NEO study thank all individuals who participated in the Netherlands Epidemiology in Obesity study, all participating general practitioners for inviting eligible participants and all research nurses for collection of the data. We thank the NEO study group, Petra Noordijk, Pat van Beelen and Ingeborg de Jonge for the coordination, lab and data management of the NEO study. The genotyping in the NEO study was supported by the Centre National de Génotypage (Paris, France), headed by Jean-Francois Deleuze. The NEO study is supported by the participating Departments, the Division and the Board of Directors of the Leiden University Medical Center, and by the Leiden University, Research Profile Area Vascular and Regenerative Medicine. Dennis Mook-Kanamori is supported by Dutch Science Organization (ZonMW-VENI Grant 916.14.023). Diana van Heemst was supported by the European Commission funded project HUMAN (Health-2013-INNOVATION-1-602757).

Pelotas Birth Cohort Study (The 1982 Pelotas Birth Cohort Study, Brazil): The 1982 Pelotas Birth Cohort Study is conducted by the Postgraduate Program in Epidemiology at Universidade Federal de Pelotas with the collaboration of the Brazilian Public Health Association (ABRASCO). From 2004 to 2013, the Wellcome Trust supported the study. The International Development Research Center, World Health Organization, Overseas Development Administration, European Union, National Support Program for Centers of Excellence (PRONEX), the Brazilian National Research Council (CNPq), and the Brazilian Ministry of Health supported previous phases of the study.

Genotyping of 1982 Pelotas Birth Cohort Study participants was supported by the Department of Science and Technology (DECIT, Ministry of Health) and National Fund for Scientific and Technological Development (FNDCT, Ministry of Science and Technology), Funding of Studies and Projects (FINEP, Ministry of Science and Technology, Brazil), Coordination of Improvement of Higher Education Personnel (CAPES, Ministry of Education, Brazil).

RS (Rotterdam Study): The Rotterdam Study is funded by Erasmus Medical Center and Erasmus University, Rotterdam, Netherlands Organization for the Health Research and Development (ZonMw), the Research Institute for Diseases in the Elderly (RIDE), the Ministry of Education, Culture and Science, the Ministry for Health, Welfare and Sports, the European Commission (DG XII), and the Municipality of Rotterdam. The authors are grateful to the study participants, the staff from the Rotterdam Study and the participating general practitioners and pharmacists.

The generation and management of GWAS genotype data for the Rotterdam Study was executed by the Human Genotyping Facility of the Genetic Laboratory of the Department of Internal Medicine, Erasmus MC, Rotterdam, The Netherlands. The GWAS datasets are supported by the Netherlands Organisation of Scientific Research NWO Investments (nr. 175.010.2005.011, 911-03-012), the Genetic Laboratory of the Department of Internal Medicine, Erasmus MC, the Research Institute for Diseases in the Elderly (014-93-015; RIDE2), the Netherlands Genomics Initiative (NGI)/Netherlands Organisation for Scientific Research (NWO) Netherlands Consortium for Healthy Aging (NCHA), project nr. 050-060-810. We thank Pascal Arp, Mila Jhamai, Marijn Verkerk, Lizbeth Herrera, Marjolein Peters and Carolina Medina-Gomez for their help in creating the GWAS database, and Karol Estrada, Yurii Aulchenko and Carolina Medina-Gomez for the creation and analysis of imputed data.

SCHS-CHD (Singapore Chinese Health Study - Coronary Heart Disease): The Singapore Chinese Health Study is supported by the National Institutes of Health, USA (RO1 CA144034 and UM1 CA182876), the nested case-control study of myocardial infarction by the Singapore National Medical Research Council (NMRC 1270/2010) and genotyping by the HUJ-CREATE Programme of the National Research Foundation, Singapore (Project Number 370062002).

SP2 (Singapore Prospective Study Program): SP2 is supported by the individual research grant and clinician scientist award schemes from the National Medical Research Council and the Biomedical Research Councils of Singapore.

WGHS (Women's Genome Health Study): The WGHS is supported by the National Heart, Lung, and Blood Institute (HL043851 and HL080467) and the National Cancer Institute (CA047988 and UM1CA182913), with collaborative scientific support and funding for genotyping provided by Amgen.

WHI (Women's Health Initiative): The WHI program is funded by the National Heart, Lung, and Blood Institute, National Institutes of Health, U.S. Department of Health and Human Services through contracts HHSN268201100046C, HHSN268201100001C, HHSN268201100002C, HHSN268201100003C, HHSN268201100004C, and HHSN271201100004C. Nora Franceschini was supported by R21-HL123677, R56-DK104806 and R01-MD012765. The authors thank the WHI investigators and staff for their dedication, and the study participants for making the program possible. A full listing of WHI investigators is at:

http://www.whi.org/researchers/Documents%20%20Write%20a%20Paper/WHI%20Investigator%20Short%20List.pdf

Supplementary Note 4

STAGE 2 STUDY ACKNOWLEDGMENTS:

AA-DHS (African American Diabetes Heart Study): The investigators acknowledge the cooperation of our Diabetes Heart Study (DHS) and AA-DHS participants. This work was supported by NIH R01 DK071891, R01 HL092301 and the General Clinical Research Center of Wake Forest School of Medicine M01-RR-07122.

Airwave (The Airwave Health Monitoring Study): We thank all participants in the Airwave Health Monitoring Study. The study is funded by the Home Office (Grant number 780-TETRA) with additional support from the National Institute for Health Research (NIHR), Imperial College Healthcare NHS Trust (ICHNT) and Imperial College Biomedical Research Centre (BRC). The study has ethical approval from the National Health Service Multi-site Research Ethics Committee (MREC/13/NW/0588). This work used computing resources provided by the MRC- funded UK MEDical Bioinformatics partnership programme (UK MED-BIO) (MR/L01632X/1). P.E. would like to acknowledge support from the Medical Research Council and Public Health England for the MRC-PHE Centre for Environment and Health (MR/L01341X/1) and from the NIHR NIHR Health Protection Research Unit in Health Impact of Environmental Hazards (HPRU-2012-10141). PE is supported by the UK Dementia Research Institute which receives its funding from UK DRI Ltd funded by the UK Medical Research Council, Alzheimer's Society and Alzheimer's Research UK; and is an Associate Director of the Health Data Research UK (HDR-UK) London Centre which receives its funding from a consortium led by the UK Medical Research Council.

BES (Beijing Eye Study): BES was supported by the National Key Laboratory Fund, Beijing, China.

BRIGHT (British Genetics of Hypertension): This work was supported by the Medical Research Council of Great Britain (grant number G9521010D) and the British Heart Foundation (grant number PG/02/128). The BRIGHT study is extremely grateful to all the patients who participated in the study and the BRIGHT nursing team. This work forms part of the research program of the National Institutes of Health Research (NIHR Cardiovascular Biomedical Research) Cardiovascular Biomedical Unit at Barts and The London, QMUL.

CFS (Cleveland Family Study): The CFS was supported by the National Institutes of Health, the National Heart, Lung, Blood Institute grant HL113338, R01HL098433, HL46380.

CoLaus (Cohorte Lausannoise): The CoLaus study was and is supported by research grants from GlaxoSmithKline, the Faculty of Biology and Medicine of Lausanne, and the Swiss National Science Foundation (grants 33CSCO-122661, 33CS30-139468 and 33CS30-148401).

DESIR (Data from an Epidemiological Study on the Insulin Resistance): The DESIR Study Group is composed of Inserm-U1018 (Paris: B. Balkau, P. Ducimetière, E. Eschwège), Inserm-U367 (Paris: F. Alhenc-Gelas), CHU d'Angers (A. Girault), Bichat Hospital (Paris: F. Fumeron, M. Marre, R. Roussel), CHU de Rennes (F. Bonnet), CNRS UMR-8199 (Lille: A. Bonnefond, P. Froguel), Medical

Examination Services (Alençon, Angers, Blois, Caen, Chartres, Chateauroux, Cholet, LeMans, Orléans and Tours), Research Institute for General Medicine (J. Cogneau), the general practitioners of the region and the Cross- Regional Institute for Health (C. Born, E. Caces, M. Cailleau, N. Copin, J.G. Moreau, F. Rakotozafy, J. Tichet, S. Vol).

The DESIR study was supported by Inserm contracts with CNAMTS, Lilly, Novartis Pharma and Sanofi-aventis, and by Inserm (Réseaux en Santé Publique, Interactions entre les déterminants de la santé, Cohortes Santé TGIR 2008), the Association Diabète Risque Vasculaire, the Fédération Française de Cardiologie, La Fondation de France, ALFEDIAM, ONIVINS, Société Francophone du Diabète, Ardix Medical, Bayer Diagnostics, Becton Dickinson, Cardionics, Merck Santé, Novo Nordisk, Pierre Fabre, Roche and Topcon.

DFTJ (Dongfeng-Tongji Cohort Study): This work was supported by grants from the Foundation of National Key Program of Research and Development of China (2016YFC0900800), the Programme of Introducing Talents of Discipline, the grants from the National Natural Science Foundation (grant NSFC-81473051, 81522040 and 81230069), and the Program for the New Century Excellent Talents in University (NCET-11-0169).

DHS (Diabetes Heart Study): The authors thank the investigators, staff, and participants of the DHS for their valuable contributions. This study was supported by the National Institutes of Health through HL67348 and HL092301.

DR's EXTRA (Dose-Responses to Exercise Training): The study was supported by grants from Ministry of Education and Culture of Finland (722 and 627; 2004-2010); Academy of Finland (102318, 104943, 123885, 211119); European Commission FP6 Integrated Project (EXGENESIS), LSHM-CT-2004-005272; City of Kuopio; Juho Vainio Foundation; Finnish Diabetes Association; Finnish Foundation for Cardiovascular Research; Kuopio University Hospital; Päivikki and Sakari Sohlberg Foundation; Social Insurance Institution of Finland 4/26/2010.

EGCUT (Estonian Genome Center - University of Tartu (Estonian Biobank)): This study was supported by EU H2020 grants 692145, 676550, 654248, Estonian Research Council Grant IUT20-60 and PUT1660, NIASC, EIT – Health and NIH-BMI Grant No: 2R01DK075787-06A1 and EU through the European Regional Development Fund (Project No. 2014-2020.4.01.15-0012 GENTRANSMED).

EPIC (European Prospective Investigation into Cancer and Nutrition)-Norfolk: The EPIC Norfolk Study is funded by Cancer Research, United Kingdom, British Heart Foundation, the Medical Research Council, the Ministry of Agriculture, Fisheries and Food, and the Europe against Cancer Programme of the Commission of the European Communities. We thank all EPIC participants and staff for their contribution to the study.

FUSION (Finland-United States Investigation of NIDDM Genetics): The FUSION study was supported by DK093757, DK072193, DK062370, and ZIA-HG000024. Genotyping was conducted at the Genetic Resources Core Facility (GRCF) at the Johns Hopkins Institute of Genetic Medicine.

GeneSTAR (Genetic Studies of Atherosclerosis Risk): [for the smoking/lipids and smoking/BP analyses] GeneSTAR was supported by National Institutes of Health grants from the National Heart,

Lung, and Blood Institute (HL49762, HL59684, HL58625, HL071025, U01 HL72518, and HL087698), National Institute of Nursing Research (NR0224103), and by a grant from the National Center for Research Resources to the Johns Hopkins General Clinical Research Center (M01-RR000052).

GLACIER (Gene x Lifestyle Interactions and Complex Traits Involved in Elevated Disease Risk): We thank the participants, health professionals and data managers involved in the Västerbotten Intervention Programme. We are also grateful to the staff of the Northern Sweden Biobank for preparing materials and to K Enqvist and T Johansson (Västerbottens County Council, Umeå, Sweden) for DNA preparation. The Västerbotten Intervention Programme is financed by Västerbotten County Council. The current study was supported by Novo Nordisk (PWF), the Swedish Research Council (PWF), the Swedish Heart Lung Foundation (PWF), the European Research Council (PWF), and the Skåne Health Authority (PWF).

GRAPHIC (Genetic Regulation of Arterial Pressure of Humans in the Community): The GRAPHIC Study was funded by the British Heart Foundation (BHF/RG/2000004). This work falls under the portfolio of research supported by the NIHR Leicester Cardiovascular Biomedical Research Unit. CPN and NJS are funded by the BHF and NJS is a NIHR Senior Investigator.

HCHS/SOL (Hispanic Community Health Study/ Study of Latinos): The baseline examination of HCHS/SOL was supported by contracts from the National Heart, Lung, and Blood Institute (NHLBI) to the University of North Carolina (N01-HC65233), University of Miami (N01-HC65234), Albert Einstein College of Medicine (N01-HC65235), Northwestern University (N01-HC65236), and San Diego State University (N01-HC65237). The National Institute on Minority Health and Health Disparities, National Institute on Deafness and Other Communication Disorders, National Institute of Dental and Craniofacial Research (NIDCR), National Institute of Diabetes and Digestive and Kidney Diseases (NIDDK), National Institute of Neurological Disorders and Stroke, and NIH Office of Dietary Supplements additionally contributed funding to HCHS/SOL. The Genetic Analysis Center at the University of Washington was supported by NHLBI and NIDCR contracts (HHSN268201300005C AM03 and MOD03). Additional analysis support was provided by 1R01DK101855-01 and 13GRNT16490017. Genotyping was also supported by National Center for Advancing Translational Sciences UL1TR000124 and NIDDK DK063491 to the Southern California Diabetes Endocrinology Research Center. This research was also supported in part by the Intramural Research Program of the NIDDK, contract no. HHSB268201200054C, and Illumina.

HRS (Health and Retirement Study): HRS is supported by the National Institute on Aging (NIA U01AG009740 and R03 AG046389). Genotyping was funded separately by NIA (RC2 AG036495, RC4 AG039029). Our genotyping was conducted by the NIH Center for Inherited Disease Research (CIDR) at Johns Hopkins University. Genotyping quality control and final preparation of the data were performed by the Genetics Coordinating Center at the University of Washington.

HyperGEN-AXIOM (Hypertension Genetic Epidemiology Network): The study was support by the National Institutes of Health, the National Heart, Lung, Blood Institute grant HL086718.

INGI-CARL (Italian Network Genetic Isolates): This study was partially supported by Regione FVG (L.26.2008) and Italian Ministry of Health (GR-2011-02349604).

INGI-FVG (Italian Network Genetic Isolates): This study was partially supported by Regione FVG (L.26.2008) and Italian Ministry of Health (GR-2011-02349604).

IRAS Family Study (Insulin Resistance Atherosclerosis Study): The IRASFS is supported by the National Heart Lung and Blood Institute (HL060944, HL061019, and HL060919). Genotyping for this study was supported by the GUARDIAN Consortium with grant support from the National Institute of Diabetes and Digestive and Kidney Diseases (NIDDK; DK085175) and in part by UL1TR000124 (CTSI) and DK063491 (DRC). The authors thank study investigators, staff, and participants for their valuable contributions.

JUPITER (Justification for the Use of Statins in Primary Prevention: An Intervention Trial Evaluating Rosuvastatin): Support for genotype data collection and collaborative genetic analysis in JUPITER was provided by Astra-Zeneca.

KORA (Cooperative Health Research in the Augsburg Region): The KORA study was initiated and financed by the Helmholtz Zentrum München – German Research Center for Environmental Health, which is funded by the German Federal Ministry of Education and Research (BMBF) and by the State of Bavaria. Furthermore, KORA research was supported within the Munich Center of Health Sciences (MC-Health), Ludwig-Maximilians-Universität, as part of LMUinnovativ.

LBC1936 (Lothian Birth Cohort 1936): We thank the LBC1936 cohort participants and team members who contributed to these studies. Phenotype collection was supported by Age UK (The Disconnected Mind project). Genotyping was funded by the BBSRC (BB/F019394/1). The work was undertaken by The University of Edinburgh Centre for Cognitive Ageing and Cognitive Epidemiology, part of the cross council Lifelong Health and Wellbeing Initiative (MR/K026992/1). Funding from the BBSRC and Medical Research Council (MRC) is gratefully acknowledged.

LifeLines (Netherlands Biobank): The Lifelines Cohort Study, and generation and management of GWAS genotype data for the Lifelines Cohort Study is supported by the Netherlands Organization of Scientific Research NWO (grant 175.010.2007.006), the Economic Structure Enhancing Fund (FES) of the Dutch government, the Ministry of Economic Affairs, the Ministry of Education, Culture and Science, the Ministry for Health, Welfare and Sports, the Northern Netherlands Collaboration of Provinces (SNN), the Province of Groningen, University Medical Center Groningen, the University of Groningen, Dutch Kidney Foundation and Dutch Diabetes Research Foundation.

The authors wish to acknowledge the services of the Lifelines Cohort Study, the contributing research centers delivering data to Lifelines, and all the study participants.

METSIM (Metabolic Syndrome In Men): The METSIM study was supported by the Academy of Finland (contract 124243), the Finnish Heart Foundation, the Finnish Diabetes Foundation, Tekes (contract 1510/31/06), and the Commission of the European Community (HEALTH-F2-2007 201681), and the US National Institutes of Health grants DK093757, DK072193, DK062370, and ZIA-

HG000024. Genotyping was conducted at the Genetic Resources Core Facility (GRCF) at the Johns Hopkins Institute of Genetic Medicine.

NESDA (Netherlands Study of Depression and Anxiety): Funding was obtained from the Netherlands Organization for Scientific Research (Geestkracht program grant 10-000-1002); the Center for Medical Systems Biology (CSMB, NWO Genomics), Biobanking and Biomolecular Resources Research Infrastructure (BBMRI-NL), VU University's Institutes for Health and Care Research (EMGO+) and Neuroscience Campus Amsterdam, University Medical Center Groningen, Leiden University Medical Center, National Institutes of Health (NIH, R01D0042157-01A, MH081802, Grand Opportunity grants 1RC2 MH089951 and 1RC2 MH089995). Part of the genotyping and analyses were funded by the Genetic Association Information Network (GAIN) of the Foundation for the National Institutes of Health.Computing was supported by BiG Grid, the Dutch e-Science Grid, which is financially supported by NWO.

PREVEND (The Prevention of REnal and Vascular ENd stage Disease study): PREVEND genetics is supported by the Dutch Kidney Foundation (Grant E033), the EU project grant GENECURE (FP-6 LSHM CT 2006 037697), the National Institutes of Health (grant 2R01LM010098), The Netherlands organization for health research and development (NWO-Groot grant 175.010.2007.006, NWO VENI grant 916.761.70, ZonMw grant 90.700.441). Niek Verweij was supported by NWO VENI (016.186.125).

RHS (Ragama Health Study): The RHS was supported by the Grant of National Center for Global Health and Medicine (NCGM).

SHEEP (Stockholm Heart Epidemiology Project): This study was supported by grants from the Swedish Research Council for Health, Working Life and Welfare (http://www.forte.se/en/), the Stockholm County Council (http://www.sll.se/om-landstinget/Information-in-English1/), the Swedish Research Council (http://www.vr.se/inenglish.4.12fff4451215cbd83e4800015152.html), the Swedish Heart and Lung Foundation (https://www.hjart-lungfonden.se/HLF/Om-Hjart-lungfonden/About-HLF/), and the Cardiovascular Programme at Karolinska Institutet (http://ki.se/en/mmk/cardiovascular-research-networks).

SWHS/SMHS (Shanghai Women's Health Study/ Shanghai Men's Health Study): We thank all the individuals who took part in these studies and all the researchers who have enabled this work to be carried out. The Shanghai Women's Health Study and the Shanghai Men's Health Study are supported by research grants UM1CA182910 and UM1CA173640 from the U.S. National Cancer Institute, respectively.

TRAILS (Tracking Adolescents' Individual Lives Survey): TRAILS (TRacking Adolescents' Individual Lives Survey) is a collaborative project involving various departments of the University Medical Center and University of Groningen, the Erasmus University Medical Center Rotterdam, the University of Utrecht, the Radboud Medical Center Nijmegen, and the Parnassia Bavo group, all in the Netherlands. TRAILS has been financially supported by grants from the Netherlands Organization for Scientific Research NWO (Medical Research Council program grant GB-MW 940-38-011; ZonMW Brainpower grant 100-001-004; ZonMw Risk Behavior and Dependence grant 60-60600-97-118;

ZonMw Culture and Health grant 261-98-710; Social Sciences Council medium-sized investment grants GB-MaGW 480-01-006 and GB-MaGW 480-07-001; Social Sciences Council project grants GB-MaGW 452-04-314 and GB-MaGW 452-06-004; NWO large-sized investment grant 175.010.2003.005; NWO Longitudinal Survey and Panel Funding 481-08-013); the Dutch Ministry of Justice (WODC), the European Science Foundation (EuroSTRESS project FP-006), Biobanking and Biomolecular Resources Research Infrastructure BBMRI-NL (CP 32), the participating universities, and Accare Center for Child and Adolescent Psychiatry. Statistical analyses were carried out on the Genetic Cluster Computer (http://www.geneticcluster.org), which is financially supported by the Netherlands Scientific Organization (NWO 480-05-003) along with a supplement from the Dutch Brain Foundation.

We are grateful to all adolescents who participated in this research and to everyone who worked on this project and made it possible.

TWINGENE (TwinGene of the Swedish Twin Registry): The Swedish Twin Registry is financially supported by Karolinska Institutet. TwinGene project received funding from the Swedish Research Council (M-2005-1112), GenomEUtwin (EU/QLRT-2001-01254; QLG2-CT-2002-01254), NIH DK U01-066134, The Swedish Foundation for Strategic Research (SSF) and the Heart and Lung foundation no. 20070481

YFS (The Cardiovascular Risk in Young Finns Study): The Young Finns Study has been financially supported by the Academy of Finland: grants 286284, 134309 (Eye), 126925, 121584, 124282, 129378 (Salve), 117787 (Gendi), and 41071 (Skidi); the Social Insurance Institution of Finland; Kuopio, Tampere and Turku University Hospital Medical Funds (grant X51001); Juho Vainio Foundation; Paavo Nurmi Foundation; Finnish Foundation for Cardiovascular Research ; Finnish Cultural Foundation; Tampere Tuberculosis Foundation; Emil Aaltonen Foundation; Yrjö Jahnsson Foundation; Signe and Ane Gyllenberg Foundation; and Diabetes Research Foundation of Finnish Diabetes Association.

The expert technical assistance in the statistical analyses by Leo-Pekka Lyytikäinen and Irina Lisinen is gratefully acknowledged.

Supplementary References

1. The ARIC Investigators. The Atherosclerosis Risk in Communities (ARIC) Study: design and objectives. *Am J Epidemiol* **129**, 687-702 (1989).
2. de Oliveira, C.M., Pereira, A.C., de Andrade, M., Soler, J.M. & Krieger, J.E. Heritability of cardiovascular risk factors in a Brazilian population: Baependi Heart Study. *BMC Med Genet* **9**, 32 (2008).
3. Friedman, G.D. et al. CARDIA: study design, recruitment, and some characteristics of the examined subjects. *J Clin Epidemiol* **41**, 1105-1116 (1988).
4. Fried, L.P. et al. The Cardiovascular Health Study: design and rationale. *Ann Epidemiol* **1**, 263-276 (1991).
5. Higgins, M. et al. NHLBI Family Heart Study: objectives and design. *Am J Epidemiol* **143**, 1219-1228 (1996).
6. FBPP Investigators. Multi-center genetic study of hypertension: The Family Blood Pressure Program (FBPP). *Hypertension* **39**, 3-9 (2002).
7. Daniels, P.R. et al. Familial aggregation of hypertension treatment and control in the Genetic Epidemiology Network of Arteriopathy (GENOA) study. *Am J Med* **116**, 676-681 (2004).
8. Wyatt, S.B. et al. A community-driven model of research participation: the Jackson Heart Study Participant Recruitment and Retention Study. *Ethn Dis* **13**, 438-455 (2003).
9. Taylor, H.A., Jr. et al. Toward resolution of cardiovascular health disparities in African Americans: design and methods of the Jackson Heart Study. *Ethn Dis* **15**, S6-4-17 (2005).
10. Fuqua, S.R. et al. Recruiting African-American research participation in the Jackson Heart Study: methods, response rates, and sample description. *Ethn Dis* **15**, S6-18-29 (2005).
11. Bild, D.E. et al. Multi-Ethnic Study of Atherosclerosis: objectives and design. *Am J Epidemiol* **156**, 871-881 (2002).
12. Victora, C.G. & Barros, F.C. Cohort profile: the 1982 Pelotas (Brazil) birth cohort study. *Int J Epidemiol* **35**, 237-242 (2006).
13. Horta, B.L. et al. Cohort Profile Update: The 1982 Pelotas (Brazil) Birth Cohort Study. *Int J Epidemiol* **44**, 441, 441a-441e (2015).
14. Nang, E.E. et al. Validity of the International Physical Activity Questionnaire and the Singapore Prospective Study Program physical activity questionnaire in a multiethnic urban Asian population. *BMC Med Res Methodol* **11**, 141 (2011).
15. Nang, E.E. et al. Is there a clear threshold for fasting plasma glucose that differentiates between those with and without neuropathy and chronic kidney disease?: the Singapore Prospective Study Program. *Am J Epidemiol* **169**, 1454-1462 (2009).
16. Tan, K.H.X. et al. Cohort Profile: The Singapore Multi-Ethnic Cohort (MEC) study. *Int J Epidemiol* (2018).
17. Hays, J. et al. The Women's Health Initiative recruitment methods and results. *Ann Epidemiol* **13**, S18-77 (2003).
18. The Women's Health Initiative Study Group. Design of the women's health initiative clinical trial and observational study. *Control Clin Trials* **19**, 61-109 (1998).
19. Elliott, P. et al. The Airwave Health Monitoring Study of police officers and staff in Great Britain: rationale, design and methods. *Environ Res* **134**, 280-285 (2014).
20. Caulfield, M. et al. Genome-wide mapping of human loci for essential hypertension. *Lancet* **361**, 2118-2123 (2003).
21. Wang, F. et al. Cohort Profile: the Dongfeng-Tongji cohort study of retired workers. *Int J Epidemiol* **42**, 731-740 (2013).
22. Scott, L.J. et al. A genome-wide association study of type 2 diabetes in Finns detects multiple susceptibility variants. *Science* **316**, 1341-1345 (2007).

23. Kurbasic, A. et al. Gene-Lifestyle Interactions in Complex Diseases: Design and Description of the GLACIER and VIKING Studies. *Curr Nutr Rep* **3**, 400-411 (2014).
24. Thomas Juster, F. & Suzman, R. Juster FT, Suzman R. An overview of the health and retirement study. *J Hum Res **40**:* S7-S56, (1995).
25. Sonnega, A. et al. Cohort Profile: the Health and Retirement Study (HRS). *Int J Epidemiol* **43**, 576-85 (2014).
26. Ridker, P.M. et al. Rosuvastatin to prevent vascular events in men and women with elevated C-reactive protein. *N Engl J Med* **359**, 2195-2207 (2008).
27. Chasman, D.I. et al. Genetic determinants of statin-induced low-density lipoprotein cholesterol reduction: the Justification for the Use of Statins in Prevention: an Intervention Trial Evaluating Rosuvastatin (JUPITER) trial. *Circ Cardiovasc Genet* **5**, 257-264 (2012).
28. Deary, I.J., Gow, A.J., Pattie, A. & Starr, J.M. Cohort profile: the Lothian Birth Cohorts of 1921 and 1936. *Int J Epidemiol* **41**, 1576-1584 (2012).
29. Scholtens, S. et al. Cohort Profile: LifeLines, a three-generation cohort study and biobank. *Int J Epidemiol* **44**, 1172-1180 (2015).
30. Stancakova, A. et al. Changes in insulin sensitivity and insulin release in relation to glycemia and glucose tolerance in 6,414 Finnish men. *Diabetes* **58**, 1212-1221 (2009).
31. Penninx, B.W. et al. The Netherlands Study of Depression and Anxiety (NESDA): rationale, objectives and methods. *Int J Methods Psychiatr Res* **17**, 121-140 (2008).
32. Hillege, H.L. et al. Urinary albumin excretion predicts cardiovascular and noncardiovascular mortality in general population. *Circulation* **106**, 1777-1782 (2002).
33. Dassanayake, A.S. et al. Prevalence and risk factors for non-alcoholic fatty liver disease among adults in an urban Sri Lankan population. *J Gastroenterol Hepatol* **24**, 1284-1288 (2009).
