# Supplementary material for: Multi-ancestry study of blood lipid levels identifies four loci interacting with physical activity
Source: Nat Commun. 2019 Jan 22;10:376. doi: 10.1038/s41467-018-08008-w (PMC6342931; doi:10.1038/s41467-018-08008-w)
Supplement: Supplementary file 3 — Description of Additional Supplementary Files [file 41467_2018_8008_MOESM3_ESM.pdf]

- **Supplementary Data 1:** Methods for assessing physical activity and the definitions of inactivity in the participating studies
- **Supplementary Data 2:** Trait distribution in Stage 1 studies
- **Supplementary Data 3:** Trait distribution in Stage 2 studies
- **Supplementary Data 4:** SNPxPA interaction results in trans-ancestry meta-analyses for HDL cholesterol for 260 previously identified HDL cholesterol loci
- **Supplementary Data 5:** SNPxPA interaction results in trans-ancestry meta-analyses for HDL cholesterol for 202 previously identified LDL cholesterol loci
- **Supplementary Data 6:** SNPxPA interaction results in trans-ancestry meta-analyses for triglyceride levels for 185 previously identified triglyceride loci
- **Supplementary Data 7:** Previously reported 260 loci associated with HDL cholesterol
- **Supplementary Data 8:** Previously reported 202 loci associated with LDL cholesterol
- **Supplementary Data 9:** Previously reported 185 loci associated with triglyceride levels
- **Supplementary Data 10:** All genome-wide significant loci in the joint test for SNP main effect and SNPxPA interaction.
